# Clinical trials and their impact on policy during COVID-19: a review

**DOI:** 10.12688/wellcomeopenres.19305.1

**Published:** 2024-01-30

**Authors:** Paul Glasziou, Sharon Sanders, Oyungerel Byambasuren, Rae Thomas, Tammy Hoffmann, Hannah Greenwood, Madeleen van der Merwe, Justin Clark

**Affiliations:** 1 IEBH, Health Science and Medicine, Bond University, Robina, Queensland, Australia

**Keywords:** COVID-19, clinical trials, adaptive trials, platform trials, pandemic, SARS-CoV-2, treatment, health policy

## Abstract

**Background:**

Of over 8,000 recorded randomised trials addressing COVID-19, around 80% were of treatments, and 17% have reported results. Approximately 1% were adaptive or platform trials, with 25 having results available, across 29 journal articles and 10 preprint articles.

**Methods:**

We conducted an extensive literature review to address four questions about COVID-19 trials, particularly the role and impact of platform/adaptive trials and lessons learned.

**Results:**

The key findings were:

*Q1. **Social value in conducting trials and uptake into policy**?* COVID-19 drug treatments varied substantially and changed considerably, with drugs found effective in definitive clinical trials replacing unproven drugs. Dexamethasone has likely saved ½-2 million lives, and was cost effective across a range of countries and populations, whereas the cost effectiveness of remdesivir is uncertain. Published economic and health system impacts of COVID-19 treatments were infrequent.

*Q2. **Issues with adaptive trial design**s.* Of the 77 platform trials registered, 6 major platform trials, with approximately 50 treatment arms, recruited ~135,000 participants with funding over $100 million.

*Q3. **Models of good practice.*** Streamlined set-up processes such as flexible and fast-track funding, ethics, and governance approvals are vital. To facilitate recruitment, simple and streamlined research processes, and pre-existing research networks to coordinate trial planning, design, conduct and practice change are crucial to success.

*Q4. **Potential conflicts to avoid?*** When treating patients through trials, balancing individual and collective rights and allocating scarce resources between healthcare and research are challenging. Tensions occur between commercial and non-commercial sectors, and academic and public health interests, such as publication and funding driven indicators and the public good.

**Conclusion:**

There is a need to (i) reduce small, repetitive, single centre trials, (ii) increase coordination to ensure robust research conducted for treatments, and (iii) a wider adoption of adaptive/platform trial designs to respond to fast-evolving evidence landscape.

## Background

The emergence of SARS-CoV-2 in November 2019, and the subsequent declaration of COVID-19 pandemic on 11 ^th^ March 2020 were quickly followed by clinical trials of treatment options to manage the disease. As part of an overview of the ethics of clinical trials during the pandemic, the WHO commissioned this work to address four key questions:

**Q1.** *What is the relationship between social value in conducting trials and uptake into policy making, especially in an emergency context?*

**Q2.** *Are there particular issues with adaptive trial designs that need to be considered in – i.e., not the ‘gold standard’, weight of evidence?*

**Q3.** *Are there models of good practice we can learn from? From past outbreaks, from COVID, more generally?*

**Q4.** *What are some of the potential conflicts we need to avoid?*

### Current COVID-19 trials landscape

To answer these questions, it is helpful to understand the current COVID-19 trials landscape, including (i) the number of trials registered, (ii) the number of trials reported, and (iii) sub-grouped by different types of treatments studied.

We compared COVID-19 trial characteristics as reported in two prominent databases that centralise COVID-related evidence from multiple sources: the Cochrane COVID-19 Study register ^1^ and the Epistemonikos L*OVE COVID-19 repository ^2^ (see Table 1).

**Table 1.** Comparison of main COVID-19 trial characteristics from the Cochrane COVID-19 Study register and the Epistemonikos L*OVE COVID-19 repository as at 22 February 2022.

Study Characteristic	Cochrane COVID-19 study register ^1^	Epistemonikos L*OVE COVID-19 repository ^2^
**Primary studies - recorded**	**115,224**	**250,412**
**Randomised trials**		
**Recorded**	**8,165**	**6,961**
**Registered**	**5,763**	**NR**
**Reported**	**1,405**	**2,145**
**Adaptive trials ^4^**		
**Recorded**	**81**	**NR**
**Registered**	**77**	**NR**
**Reported**	**25**	**NR**
**Trial focus ^1^**		
**Treatment/management**	**4,034**	**6,961** ^2^
Prevention	1,137	^2^
Health services	434	NR
Diagnostic/prognostic	90	616
Transmission	56	91 ^3^
Epidemiology	20	585
Mechanism	7	NR
Other	675	NR
**Database characteristics**		
Established	April 2020	June 2020
Data sources	6 sources - trial registries (ClinicalTrials.gov, ICTRP), pre-print servers (medRxiv), and publication databases (PubMed, Embase and CENTRAL)	40 sources - trial registries (e.g. ICTRP, ISRCTN, ClinicalTrials.gov, Chinese Clinical Trial registry) and preprint servers (e.g. medRxiv, bioRxiv, SciELO), and publication databases (e.g. PubMed, EMBASE, CINAHL, PsycINFO),
Recording style	Study-based: all references associated with the same study (e.g., pre-prints, registry records, manuscripts etc) are linked together as one record	Record-based: all references associated with the same study are entered as separate records and linked together via a ‘publication thread’
Search strategy (appendix 1)	Peer-reviewed	Not peer-reviewed
Search conducts	Machine learning facilitate initial screening, followed by manual screening by specialists	In addition to database searches, manual searches of evidence syntheses, social media, conferences, and press releases are conducted

Updated 22/02/2022; ^1^ Not mutually exclusive, classifications as reported in Cochrane, different classifications used in Epistemonikos; ^2^ Treatment, management and prevention reported jointly in Epistemonikos.; ^3^ called aetiology in Epistemonikos; ^4^ Updated 31/03/2022

As summarised in Table 1, Epistemonikos has more primary studies reported than Cochrane, however, Cochrane has more recorded randomised trials than Epistemonikos. Although Epistemonikos does not reported whether a trial is registered, there is a disparity in Cochrane between those recorded in the database and those registered to a trial registry. Of the recorded randomised trials, 17% in Cochrane registry have reported results compared to 31% in Epistemonikos. Intervention or treatment type studies make up most of the recorded studies in both registries (>80%). Cochrane reports 81 adaptive or platform trials. Only 77 are registered through trial registers, meaning 4 trials may be unregistered. Of the 81 recorded adaptive/platform trials, 25 have results available, across 29 journal articles and 10 preprint articles.

**Figure 1.**
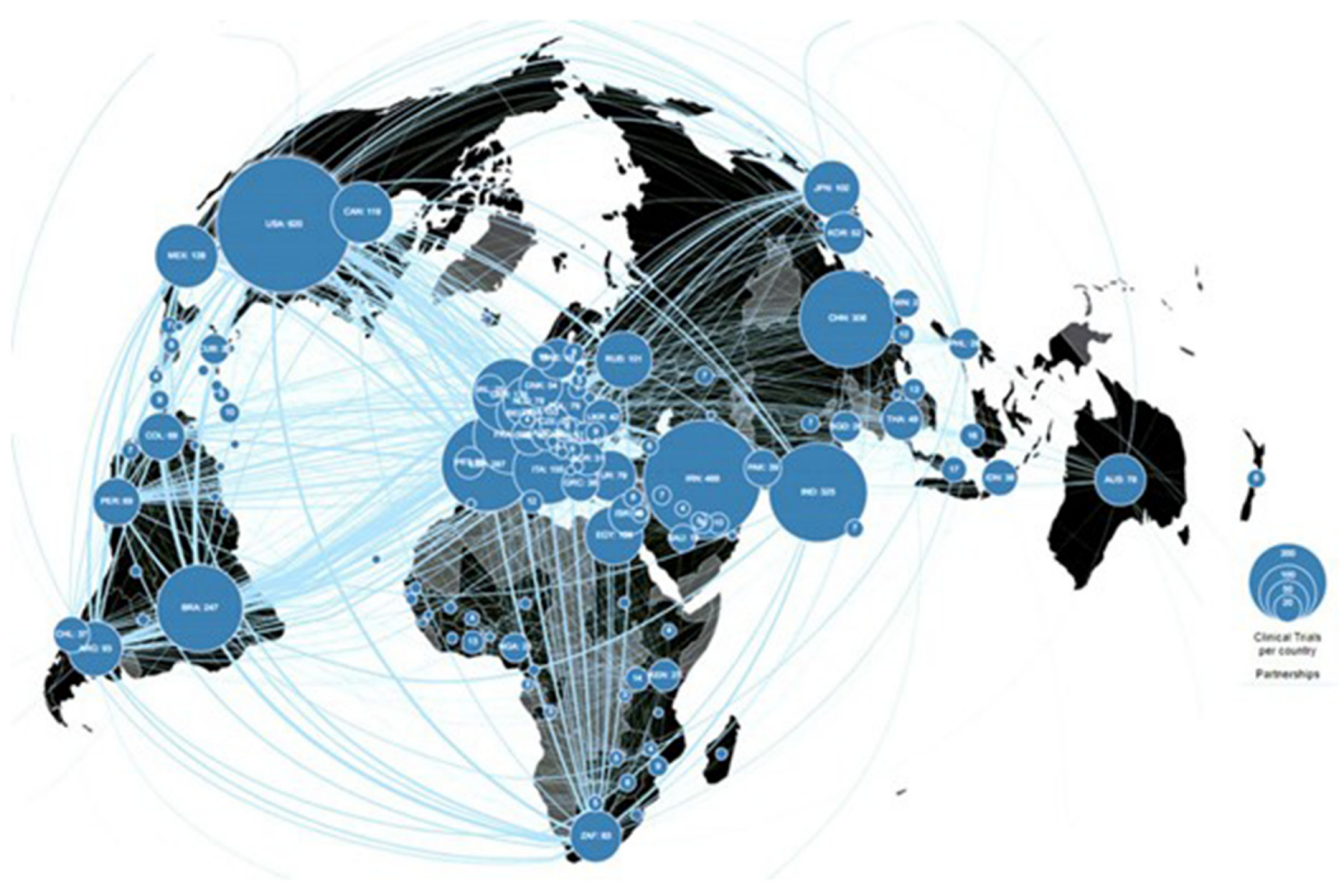
COVID-19 Living evidence synthesis project map of COVID-19 trials registered in the WHO trials registry at March 2, 2022. (from https://covid-nma.com/dataviz/) (Reproduced with permission from website producers).

Without further analysis of the trials within the databases, it is not feasible to report studies by population and country subgroups. This is somewhat captured by COVID-NMA living evidence synthesis project which provides a living map of COVID-19 trials and living synthesis of reported studies that are registered in the WHO clinical trial registry ( Figure 1). The top 5 countries with the most trials registered are: United States, China, India, Iran, and Spain, and collaboration across countries is apparent.

### Other estimates

There have been numerous articles summarising the COVID-19 trials landscape since the beginning of the pandemic. We summarise some example articles in Table 2, however, it is not a comprehensive list, and a systematic search was not conducted. Due to rapidly evolving nature of evidence in this area, we prioritised articles published in 2021 and 2022 but included some highly relevant articles from 2020.

**Table 2.** Other estimates of COVID-19 trial landscape.

Article	Study Description	Key Findings
He *et al.* (2021) ^3^	An analysis of ClinicalTrials.gov characterising COVID-19 studies that aims to understand the landscape of COVID-19 research and identify issues with eligibility and generalisability	•3765 COVID-19 studies (2295 intervention; 1470 observational) •Most studies included older adults but did not exclude those with chronic conditions •Known risk factors (e.g., diabetes, hypertension, pregnancy) were under-represented
Sacks *et al.* (2022) ^4^	A cross-sectional study of all registered randomised clinical trials for the treatment of COVID-19 in the USA, with analysis of descriptive characteristics and statistical power	•200 ongoing or completed randomised trials for treatment of people with COVID-19 as at August 2020 • 44% single centre, 32% unblinded, 40% industry sponsored • Common treatments: monoclonal antibodies, antivirals, hydroxychloroquine -> in late-phase trials, power less than 25% to detect RRR 20% in mortality • Highlights need for national, coordinated trial infrastructure to increase power to detect risk reduction on key outcomes
Seidler *et al.* (2021) ^5^	A perspective paper on the landscape of clinical trials in Australia and the extent that Australian researchers have engaged in global coordination and collaboration	•Research was rapidly scaled-up in Australia during COVID-19 (68 COVID-19 and related trials) but infrastructure that enables rapid collaboration, uptake of adaptive methodologies and data sharing is needed • Merits and risks of rapid scale up considered, with proposed solutions including: protocol development to fast-track in emergencies, collaboration to pool data and results, improve use adaptive designs to respond to rapid-evolving evidence landscape
Carracedo (2021) ^6^	Analysis of trials for COVID-19 treatment and prevention in LMIC countries including Latin America/Caribbean to assess challenges and inform recommendations to ensure meaningful evidence generation.	• Of 5213 COVID-19 studies registered, 206 interventional studies (141 randomised; 88% all trials treatment focus) were conducted in this region to August 2020 • Trend towards small, repetitive, non-rigorous studies that do not yield good safety/efficacy evidence (e.g. 27 single country trials for convalescent plasma with <100 participants) • Issues in the region include small sample, no control groups, duplication of research, lack of multicentre studies/collaboration, need for national research systems
Huang (2020) ^7^	Evaluations of the characteristics (study design, sample, outcomes and interventions) of COVID-19 intervention clinical trials registered in China	• 262 intervention clinical trials registered at March 2020 • 13% 1 arm, 69% 2 arm, 18% 3+ arms; 76% randomised, 9% double blind, 60% included <100 participants; 81% in participants with mild/moderate illness
Nguyen (2021) ^8^	To describe the planning of RCTs during early stages of COVID-19 and create a living map visualising COVID- 19 trials	• At August 2020, 1,568 trials were registered globally; 85% evaluated treatment, 14% prevention. • Over 254 trials assess hydroxychloroquine, generating competition for scarce funding resources and impairing recruitment for other potential treatment • Trend of small sample size with redundancy in research questions, and trials mostly single-centre
Honarmand (2021) ^9^	To describe characteristics of COVID- 19 trials to prevent or treat and examine association between risk of bias (RoB; randomisation, single versus multicentre, funding and sample) and likelihood of a significant effect Recommendation: improve coordination and planning of research to enhance robustness of methodology and reliability of results	• Of the 91 RCTs included, 44% were single centre, 25% had sample size <50, 31% were industry funded and 82% had high or probably high RoB, 42% reported significant results • RoB due to randomisation and centre status (single centre) trials was associated with increased likelihood of finding significant results, independent of sample size and funding • Trial characteristics, particularly those related to RoB contribute to low quality evidence which may cause harm and direct attention/resources away from effective interventions • RoB should be considered by researchers and funders when designing RCTs, and collaboration in multicentre trials should be encouraged to enhance generalisability and robustness of research

RRR = relative risk reduction; RoB = risk of bias; RCT/s = randomised controlled trial/s

Common issues and lessons emerge from these articles such as the need to reduce small, repetitive, single centre trials, increase coordination across countries and institutions to ensure robust research conducted for treatments, and push towards a wider adoption of adaptive/platform trial designs to respond to fast-evolving evidence landscape

## Q1. Social value of trials

**Q1. Clear relationship between social value in conducting trials and uptake into policy making, especially in an emergency context**

**Key Findings - Social Value of Trials.**

•   COVID-19 healthcare sites, regions, and countries.

•   Treatment of hospitalized COVID-19 patients has changed considerably over the pandemic, with unproven drugs generally being replaced by drugs found effective in definitive clinical trials.

•   Economic and health system impacts of definitively tested drugs used for the treatment of COVID-19 have been infrequent, and largely limited to dexamethasone and remdesivir.

•   Dexamethasone has been found to be cost effective across a range of countries and populations, whereas the cost effectiveness of remdesivir is uncertain, with estimates ranging from cost saving to being prohibitively expensive.

•   The early clear results for dexamethasone’s effectiveness has likely saved ½-2 million lives.

The social value of health-related research refers to the importance of the knowledge that a study is likely to produce ^10^. The information produced by a study may be important because it is directly relevant for understanding or intervening on a health problem or because of its contribution to further research likely to promote individual or public health. For a study to be ethically permissible, the social value of a study must be sufficient to justify the risks costs and burdens to participating individuals.

### Aim and background

We aimed to assess the social value of clinical trials (particularly those from large platform trials) of treatments for early and advanced COVID-19 disease that were conducted during the pandemic by:

**A.**   Identifying and describing literature examining the trends in usage of COVID-19 drug therapies for which definitive clinical trial results became available during the pandemic, and

**B.**   Identifying and describing literature evaluating the health and economic impacts of clinical trials of drug therapies with definitive results.

### A. Patterns of care related to clinical trial results

***Methods***

We searched PubMed, Embase, Web of Science and Europe PMC (limited to preprints) for studies reporting patterns of COVID-19 drug therapy usage over time (for full search see Appendix 1). We also searched for reports of usage in selected national health agencies: the UK Health Security Agency (UKHSA), the Centers for Disease Control and Prevention (CDC) National Center for Health Statistics, and the Canadian Institute for Health Information. We identified studies that reported the use of disease-modifying drugs for early and advanced COVID-19 where their effectiveness was definitively determined in clinical trials, and their use (or not) recommended by the National COVID- 19 Clinical Evidence Taskforce ^11^ listed in Table 3.

**Table 3.** Drug and drug combinations recommended and not recommended for treatment of COVID- 19 by the National COVID-19 Clinical Evidence Taskforce (list obtained 22 Feb 2022).

Recommended	Not recommended
Budesonide Casirivimab plus imdevimab Molnupiravir Nirmatelvir plus ritonavir Corticosteroids Other immunomodulating drugs (Baracitinib, Sarilumab, Tocilizumab) Sotrovimab Tixagevimab plus cilgavimab Remdesivir	Aspirin Azithromycin Colchicine Convalescent plasma Hydroxychloroquine Hydroxychloroquine plus azithromycin Interferon β-1a Interferon β-1a plus lopinavir-ritonavir Lopinavir-ritonavir

Relevant studies were longitudinal assessments of drug usage that included the dates at which definitive trial results became available. Studies evaluating the use of a definitively tested drug at a single point in time before and or during the pandemic, or over a period of time before definitive trial results became available were excluded. Studies of usage as measured by drug treatment received, prescriptions dispensed or claims data in any country in single or multiple healthcare providers or from administrative data were considered relevant. The characteristics of relevant studies were tabulated according to location and setting, study participants, dates over which data was collected and the definitively tested drug treatments with reported usage (Appendix 2 Table).

We selected studies or study data to illustrate trends in usage based on representativeness of the data globally or by country, the data source, amount, and time over which data was collected and the study population. Data were extracted from original figures reported in the studies using WebPlotDigitizer ^12^. Using these data, we created figures showing drug usage globally and in the US, superimposed with key events related to definitive trials including press statements, preprints or publications. The dates of key events related to the trial were obtained from the National COVID-19 Clinical Evidence Taskforce living guidelines and the RECOVERY trial ^13^ and WHO COVID-19 Solidarity Therapeutics trial ^14^ websites.

***Results***

We screened 1787 records and identified 37 reports of 33 studies reporting the use of definitively tested drug treatments for COVID-19 over the time trial results became available. Searches for unpublished reports of usage did not identify any other relevant studies. The studies reported usage in single healthcare centres, across cities and regions, states and countries. Studies reported actual usage in hospitalised and ambulatory patients or reported usage as implied by dispensing data.

**Figure 2.**
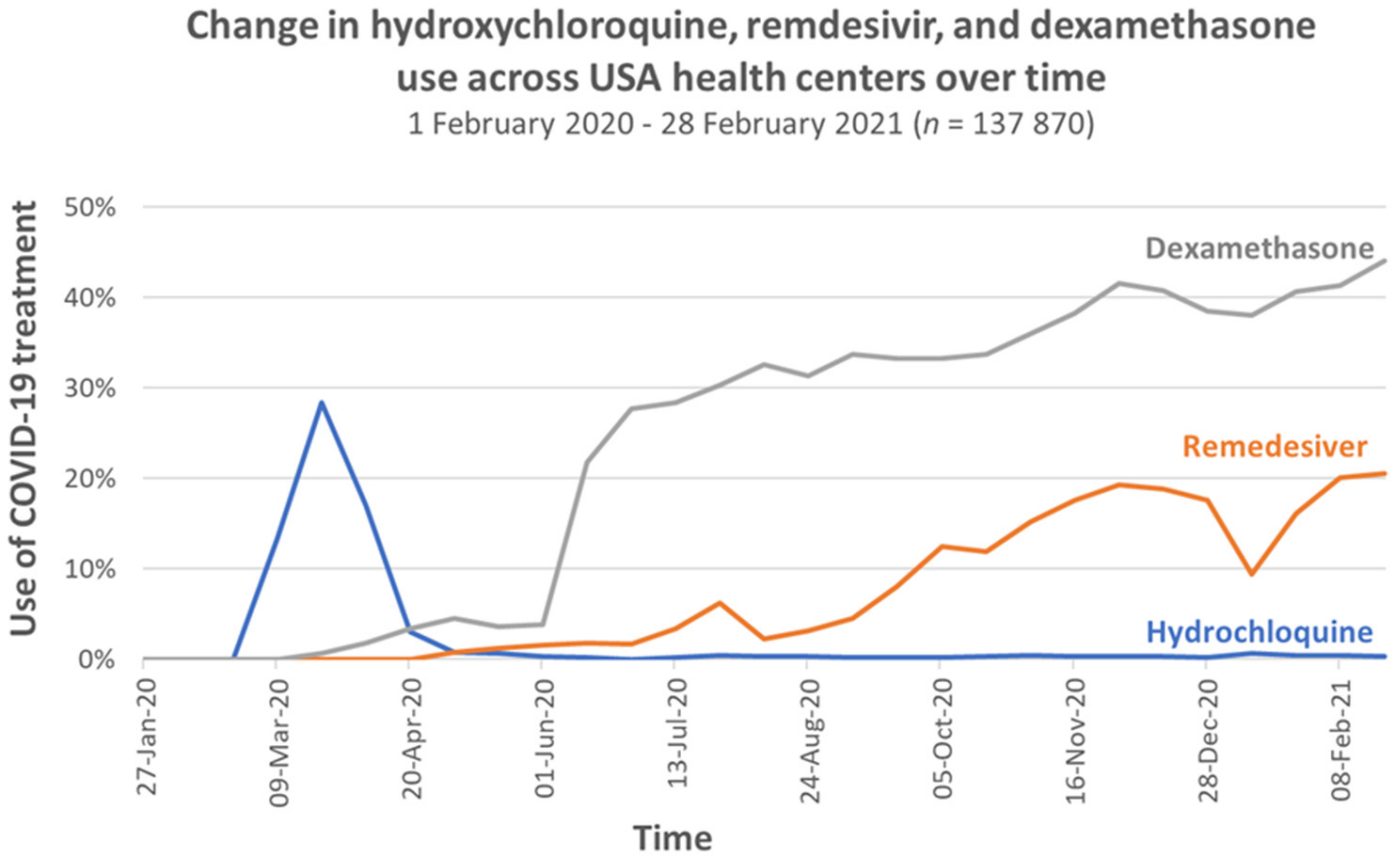
Variation in hydroxychloroquine, remdesivir and dexamethasone use across health centres in the US between February 2020 to February 2021 (Redrawn from data from Mehta *et al*., 2021 Figure 2, 15).

**Figure 3.**
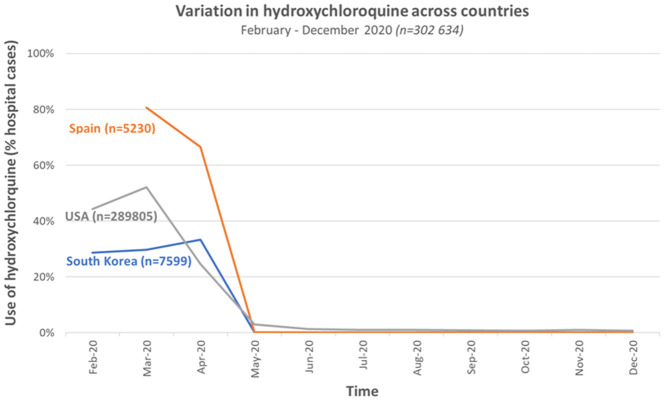
Variation in hydroxychloroquine across countries and centres between February and December 2020 (Redrawn from data in Prats-Uribe *et al*., 2021, 17).

Studies reporting usage in multiple sites showed wide variation in use across centres, states and countries, and over time. This variation is demonstrated in Figure 2 and Figure 3 below. Data on the use of hydroxychloroquine, remdesivir and dexamethasone between January 2020 and February 2021 from 43 health systems in the US ^15^ shows wide variation ( Figure 2) in hydroxychloroquine use across centres in March 2020 but reduced variation by late April 2020. It shows substantial variation in Remdesivir use across centres which persisted from May 2020 to February 2021, demonstrating how rapidly new important research evidence can be incorporated in practice ^16^. Figure 3 reports on the use of hydroxychloroquine among hospitalised patients with COVID-19 between February and December 2020 in South Korea, Spain and multiple datasets in the US ^17^. Usage of hydroxychloroquine ranged from approximately 30% in South Korea to approximately 60% in the US and 85% in Spain during the month of March 2020.

**Figure 4.**
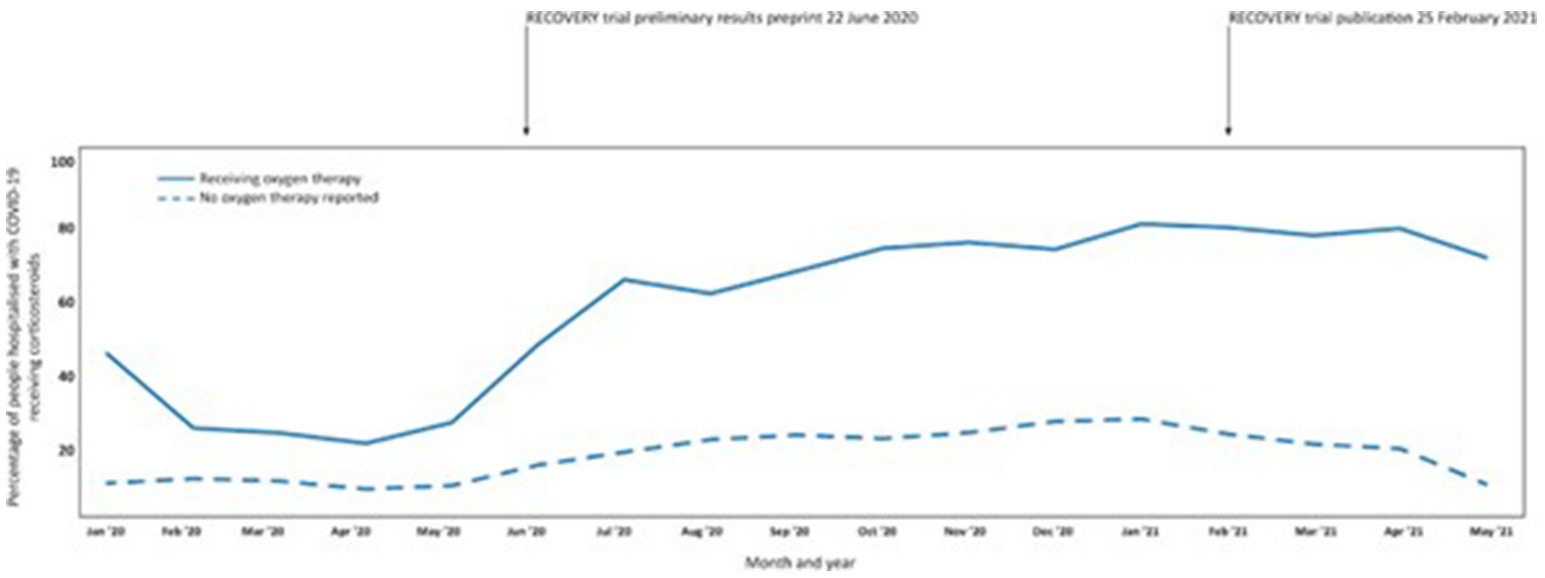
Time trends in corticosteroid use in 439,922 people admitted to hospital with COVID-19 at 720 sites in 49 countries who did and did not receive oxygen therapy between January 2020 and May 2021 (data adapted from ISARIC Clinical Characterisation Group, 2021 Supplementary figure 11 ^18^.

Trends in usage of corticosteroids across 720 sites in 49 countries between Jan 2020 and May 2021 ( Figure 4) shows use of dexamethasone was low during the first months of the pandemic. approximately 25–30% of people requiring oxygen therapy treated with dexamethasone and approximately 10% of people with no reported use of oxygen treated. Release of preliminary trial results from the RECOVERY trial – a large UK platform trial - showing a reduction in mortality with dexamethasone use, was followed by a rapid increased use of corticosteroids in patients receiving oxygen therapy. Use of corticosteroids in this group stabilised at approximately 70% in May 2021. There was a downward trend in use of dexamethasone among hospitalised people not requiring oxygen therapy coinciding with the publication of the RECOVERY trial in February 2021, which found no difference in mortality with dexamethasone among those receiving no respiratory support at randomisation.

**Figure 5.**
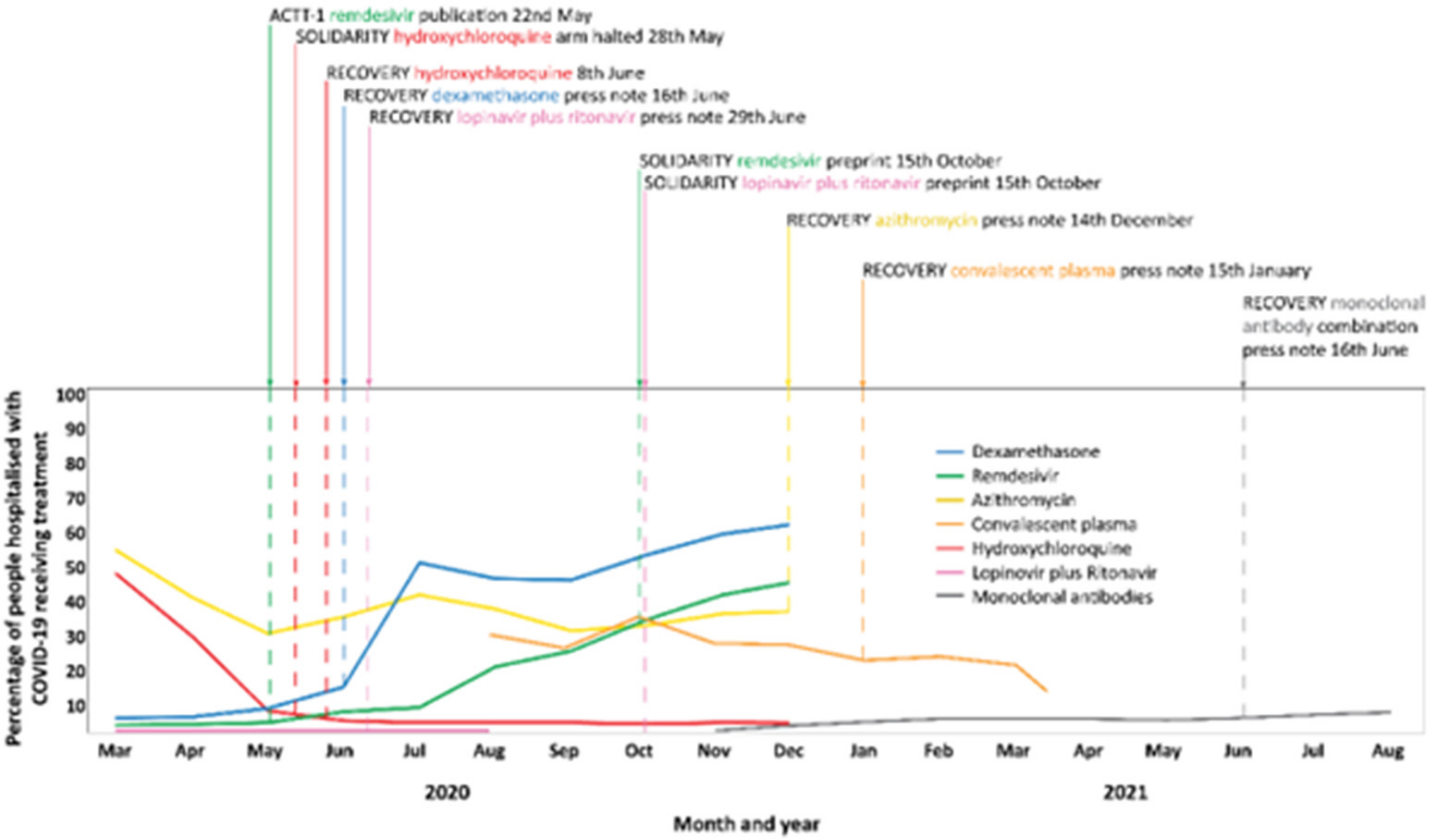
Time trends in use of definitively tested drug treatments in people hospitalized with COVID- 19 in the US between March 2020 and August 2021 (adapted from data presented in Weckstein *et al*., 2021 ^19^, Casadevall *et al*, 2021 ^20^, Prats-Uribe *et al*., 2021 ^17^ and Wiltz *et al*., 2022 ^21^). Data on time trends in use of dexamethasone, remdesivir, azithromycin and hydroxychloroquine adapted from data presented among 85,970 people in Weckstein *et al*., 2021 Figure 1 ^19^. Data on time trends in use of combined lopinavir and ritonavir adapted from data presented in Prats-Uribe *et al*., 2021 Figure 6. The data from the largest data base of 77,853 people (IQVIA Hospital CDM) were used. Data on time trends in use of convalescent plasma adapted from data presented in Casadevall *et al*., 2021 Figure 2, 20. In this study usage was inferred from the distribution of plasma units to hospitals. Data on time trends in use of monoclonal antibodies among 805,276 people adapted from Wiltz *et al*., 2022 Figure ^21^. Only data from 387,403 people of non-Hispanic ethnicity are presented.

Trends in usage of hospital treatments in the US between March 2020 and August 2021 ( Figure 5) shows that hydroxychloroquine decreased rapidly from almost 50% of patients in March 2020 to approximately 7% in May when the WHO Solidarity trial halted its hydroxychloroquine arm due to safety concerns. In December 2020 following the release of results from the RECOVERY trial which showed a lack of effectiveness of hydroxychloroquine use further dropped to 3-4%. In contrast, use of dexamethasone increased sharply in June 2020, coinciding with a press note from the RECOVERY trial indicating a reduction in mortality with dexamethasone use. Remdesivir use increased gradually from approximately 5% of patients in May 2020 following publication of the ACTT-1 platform trial which showed accelerated recovery from advanced COVID-19 with remdesivir, to approximately 35% at the time results of the WHO Solidarity trial were released in October 2020. The WHO Solidarity trial preprint showed no effect of remdesivir on mortality, ventilation or hospital stay in people admitted to hospital. However, use of remdesivir continued to increase to approximately 45% at the end of data collection in December 2020. Azithromycin use ranged from 35 to 55% between March and December 2020. There is no data on its usage after release of the RECOVERY trial results on December 14, 2020 which showed no effect of azithromycin on mortality, risk progression or hospital length of stay. Convalescent plasma use has been slowly declining from approximately 30% in November 2020 to 20% in March 2021 with a rapid decrease in use through to April 2021. The preliminary results of the RECOVERY trial in January 2021 suggested no benefit of convalescent plasma on mortality. Use of monoclonal antibodies has been stable at approximately 3-5% through the first 6 months of 2021 with a small but steady increase to almost 10% between June and August 2021 following the release of the RECOVERY trial which demonstrated reduced risk of death in hospitalised patients with severe COVID-19 who had not mounted a natural antibody response of their own.

***Discussion***

The data here demonstrated an aspect of the social value of trials – by showing trends in usage relate to the findings of definitive trials (more use of effective ones and reduced use of ineffective ones) – that are likely to have improved health, but implementation of findings varied, with uptake into practice varying from rapid for a few studies, but slow and incomplete for others.

Over the pandemic considerable changes occurred in treatment of hospitalised COVID-19 patients, with unproven treatments generally being replaced by drugs found to be effective in definitive clinical trials. Over 2020, dexamethasone and remdesivir surpassed use of hydroxychloroquine with changes in prescribing aligning with the release of the findings of large platform trials such as RECOVERY, WHO Solidarity and ACTT-1. However, there has been substantial variation in use geographically across centres, states, and countries. Variation was largest for Remdesivir where the definitive trials have shown a reduction in hospital length of stay but not mortality. There was also large variation in use of drugs such as hydroxychloroquine, prior to the availability of definitive trial results. The drivers of this variation are potentially multiple, including differences in patient case-mix, local media coverage and political stance about a drug, drug availability and regulatory decisions made in the face of an evolving evidence base.

Though change in usage has generally coincided with the availability of definitive trial results, implementation of trial findings may be slow and incomplete and the value of credible trial findings therefore limited. Novel approaches may be required to overcome this state of affairs in the context of a pandemic. One such approach is the Learning Health System approach adopted by a healthcare system in one US state which comprised rapid implementation of guideline changes based on continuous evidence evaluation and review of real time health system data via live electronic medical record forcing functions ^22^. Rapid, systemwide practice in line with findings of definitive trials of dexamethasone, remdesivir, hydroxychloroquine and tocilizumab was demonstrated (Appendix 2, Figures A - D).

The data analysed here demonstrate some aspects of the social value of trials – by showing trends in usage relate to the findings of definitive trials (more use of effective ones and reduced use of bad ones) – that are likely to have improved health, but implementation of findings into practice slow and incomplete.

### B. Health and economic impacts of clinical trials with definitive results

***Methods***

We searched PubMed, Embase, Web of Science and Europe PMC (limited to preprints) for studies reporting on the health or economic impacts of drugs used for the treatment of COVID-19 that had had their effectiveness definitively determined in a clinical trial ( Table 3). For full search strings see Appendix 1. Relevant studies were full economic evaluations comparing both the costs and health outcomes of drug treatment options for COVID-19, studies that compared the costs of two or more interventions of equal effectiveness and studies reporting admissions, ICU usage or deaths potentially avoided or caused by use of drug treatments found to be effective or ineffective in definitive clinical trials.

***Results***

During screening of database searches we identified a living systematic review of economic evaluations of drug treatments for COVID-19 ^23^. This living review included published and unpublished economic analyses found through searches of databases and model repositories up to mid-July 2021. We therefore included studies of definitively tested drugs included in this review and screened our search results for studies published subsequent to mid-July 2021.

We screened 2216 records and identified 17 reports of 15 studies evaluating the economic value or health system impacts of drugs for the treatment of COVID-19 evaluated in definitive clinical trials. Details of these studies are tabulated in Appendix 3. Half of these studies were conducted in the US or UK with the remainder in Italy, Germany, Turkey, Mexico, South Africa, and Iran in a range of mild to severely ill or mixed severity of COVID-19 populations. Seven studies were full economic evaluations of remdesivir +/- standard of care (which may or may not include treatment with steroids) compared to standard of care alone, 1 study evaluated dexamethasone alone and 4 studies evaluated both remdesivir and dexamethasone. In these studies, efficacy data were from adaptive platform trials (RECOVERY, WHO Solidarity, ACTT-1) or living systematic reviews of randomised trials.

The RECOVERY dexamethasone result in June 2020 is likely to have saved many lives. One estimate is that between July to December 2020, use of dexamethasone save 12,000 lives in the UK alone, and 650,000 lives globally. However, there is considerable uncertainty to this estimate ranging from 130,000 to 1,4000,000 depending on assumptions and statistical uncertainty around the mortality reduction. If the lives saved estimate was correct for 2021 also, that would represent a further 1,300,000 lives saved. Given the accessibility and low cost of dexamethasone, this was an important global result.

**Figure 6.**
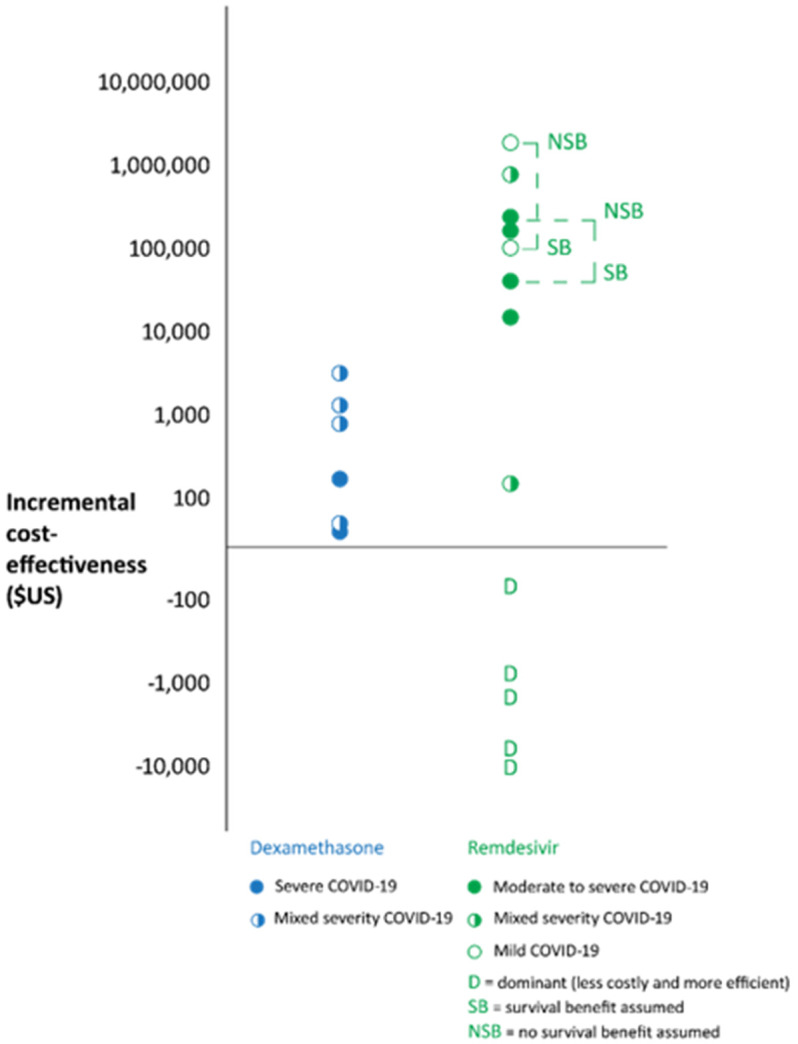
Incremental cost-effectiveness of Dexamethasone and Remdesivir. Data from one study of monoclonal antibodies was not plotted as cost-effectiveness was found to vary from cost-saving to $22671/quality adjusted life year for different ages and hospitalisation risks (Jovanski *et al*., 2022 ^24^). Data plotted was adapted from Aguas *et al*., 2021 ^25^, Jo *et al*., 2021 ^26^, Carta *et al*., 2021 ^27^, Congley *et al*., 2021 ^28^, Whittington *et al*., 2022 ^29^, I.C.E.R 2021 ^30^, Sun *et al*., 2021 ^31^, Rafia *et al*., 2022 ^32^, Ponce-de-Leon *et al*., 2022 ^33^, Oksuz *et al*., 2021 ^34^, and Gholamhossein *et al*., 2021 ^35^.

The incremental cost effectiveness of dexamethasone ranged from $174/death avoided in an ICU population in South Africa to $5208/quality adjusted life year in a hypothetical cohort of hospitalised patients with mixed severity COVID-19 (Figure). Remdesivir was found to range from being less costly and more effective & efficient (that is “dominant” over standard of care) to being expensive with an incremental cost effectiveness of up to $1,847,000 per quality adjusted life year when no survival benefit is assumed ( Figure 6).

***Discussion***

Dexamethasone was found to have saved between half a million to 2 million lives, and to accessible and cost effective across range of countries/populations. For Remdesivir the effectiveness and cost- effectiveness is much less certain with a wide range of estimates reflecting differences in cost, length of time in hospital, whether there is an effect on survival.

A notable finding is the paucity of information on global uptake of evidence: the reduction in usage of ineffective treatments and any increase in usage of effective treatments. The majority of studies arose from the US, but even those there were limited to specific health care groupings.

## Q2. Adaptive trials

**Q2. Are there particular issues with adaptive/platform trial designs that need to be considered – i.e. not the ‘gold standard’, weight of evidence?**

**Key findings - COVID-19 platform trials.**

•   Since the beginning of pandemic 77 platform trials have been registered, which is less than 2% of the more than 5,000 registered trials.

•   Six major platform trials have recruited ~135,000 participants with over $100 million in funding

•   Approximately 50 arms of treatments been opened of which half have been closed

### Background and aims

**Figure 7.**
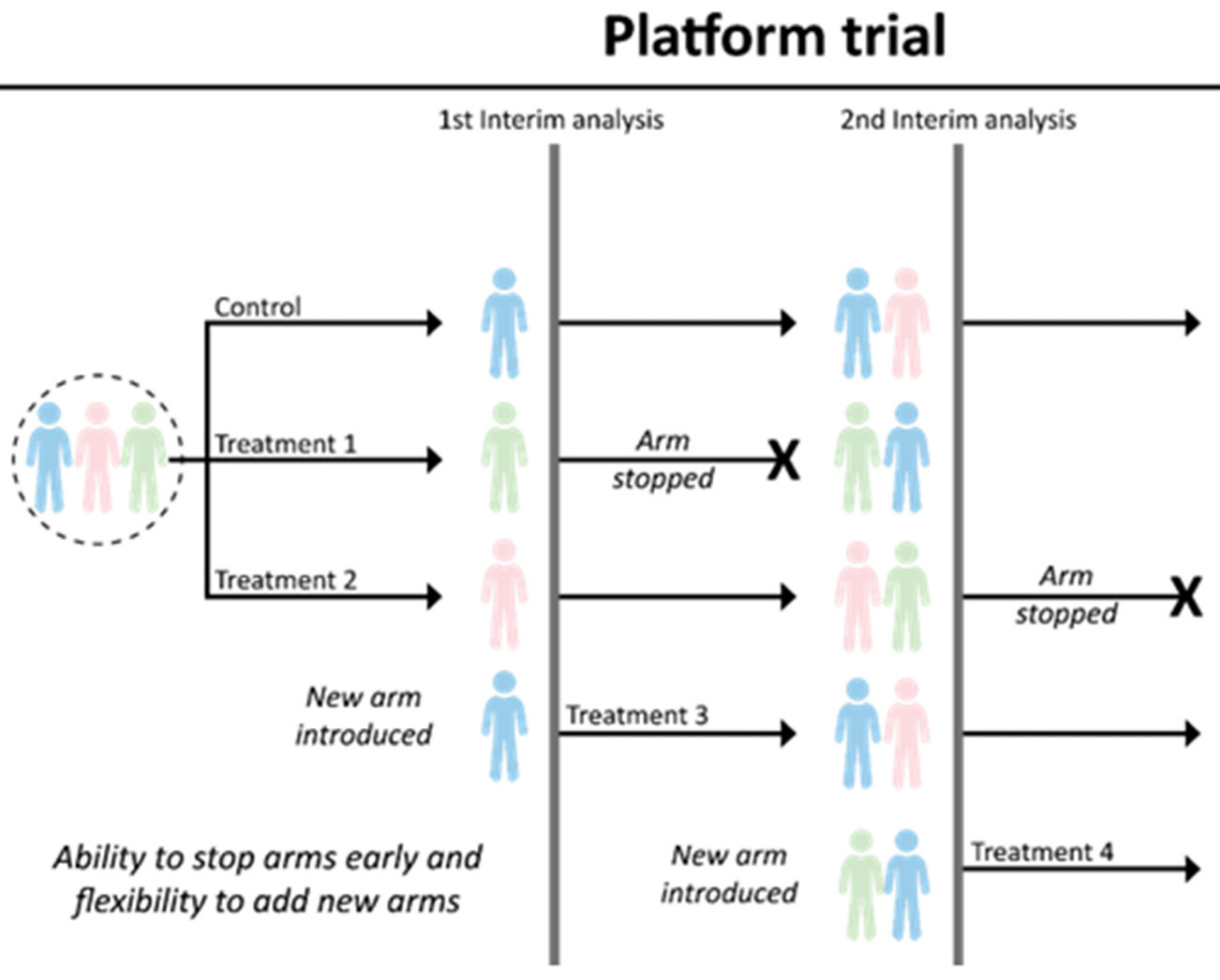
An illustration of a platform trial (Adapted from Park *et al*. ^39^). This example starts with two treatment arms plus the common control arm that consists of standard of care. There are two interim analyses planned. At the first interim analysis, intervention 1 is dropped while a new arm (treatment 3) is introduced into the platform. Another treatment (treatment 4) is introduced after the second interim analysis. Treatment 2 finishes enrolment and undergoes its planned final analysis. This hypothetical trial perpetually continues with the control arm and treatment 3 and treatment 4.

Platform and adaptive trials usually involve several arms, with some arms stopping and other arms added during the conduct of the trial ( Figure 7). Such ongoing trials which adapt to new information require a common protocol and clinical network to enable the ongoing changes.

The COVID-19 pandemic led to an unprecedented level of collaboration between researchers, industry, funding bodies, and regulators to urgently find effective treatments, and adaptive trials are one example of successful outcomes of such collaboration ^36, 37^. During the beginning of the COVID-19 pandemic, the Pan American Health Organisation (PAHO) recommended the formation of a research ethics committee to avoid duplication of clinical trials, tighter deadlines for ethical and regulatory processes, and infrastructure for efficient and safe communication between vaccine developers, academia, and government channels have been identified ^38^.

Adaptive or platform trials are “disease-focused” rather than “intervention-focused” because they allow for more efficient evaluation of multiple interventions in a perpetual manner ( Figure 7) ^39^. Such ongoing trials, which adapt to new information and new treatment options, require a standard protocol and clinical network to enable the ongoing changes.

We aimed to:

A.   estimate (i) the number of adaptive trials registered, and (ii) the number reported with any results, and

B.   summarise the recruitment, arms, findings, and issues in the key 5 to 10 platform trials including RECOVERY, SOLIDARITY, REMAP-CAPS, PRINCIPLE, TOGETHER, and PANORAMIC trials. The numbers of patients recruited will be compared with the total numbers recruited in all trials of COVID-19.

### A. Estimate of adaptive trials and reporting

***Methods***

During the preliminary search, we identified a rapid review by Vanderbeek *et al.* that reviewed 58 platform trials on COVID-19 pandemic ^40^. They have searched PubMed, ClinicalTrials.gov, and Cytel COVID-19 Clinical Trials Tracker datasets until 22 June, 2021. We summarised this review to identify issues associated with platform trial designs and cross-check their findings against other databases of adaptive trials.

***Results***

The rapid review by Vanderbeek *et al.* (2022) screened more than 2000 potential articles to identify and analyse 58 platform trials on COVID-19 prevention and treatments that were registered between January 2020 and May 2021 ^40^. Due to the lack of consensus in platform trial definition and classification, the authors chose to include trials that either self-identified as platform trials or provided evidence of adding arms during the study.

*Summary of findings from Vanderbeek *et al*., 2022. ^40^*

•   Nearly all the trials were publicly funded and ongoing.

•   Details of the trials were often incomplete or not regularly updated to ascertain many characteristics.

•   Half of the trials have made their full protocols publicly accessible.

•   A median of 3 arms have been added and 3 dropped. RECOVERY trial ^13^ has the greatest number of arms added (ten) and closed (eight).

•   In addition to adding arms, majority of the trials (84%) planned for adaptive features such as futility stopping, early efficacy stopping, sample size reassessment, and adaptive randomization.

•   A little over half of all trials (31/58, 53%) stated an intention to share individual patient data (IPD), but actual sharing rates are likely to be lower.

•   A third of the trials (36%) stated to use Bayesian methods as opposed to frequentist analysis.

### B. Summarise key trial platforms

***Methods***

We summarised 6 platform trials and 2 large trials. We extracted trial-specific data such as the number of intervention arms, number of patients recruited, country, and cost of the study (we contacted the study authors as cost-related information was not readily reported). The numbers of patients recruited were compared with the total numbers recruited in all trials of COVID-19.

***Results***

The following six platform trials have recruited more than 100,000 patients globally and raised over 100 million dollars to test the efficacy of various groups of potential treatments for COVID-19 ( Table 4). Early successes from these trials include identification of benefit of dexamethasone in reducing mortality and timely establishment of inefficacy of hydroxychloroquine, ivermectin, and convalescent plasma. Across all platform trials, approximately 50 arms were added, and more than half have been stopped since Feb 2020. Study cost estimates reported below were obtained by contacting study authors, but many could not be found readily.

**Table 4.** Summary of the key platform trials.

Trial name	N (Patients)	Arms (Open, closed)	Countries	Funders and sponsors	Drugs studied	Approx. costs
**Platform trials**						
REMAP-CAP ^43^	9,742	10 7	Initially, 50 ICUs in 13 countries on 3 continents. Now over 300 sites across 21 countries *	European Union, NHMRC, HRC, CIHR-SPOR, and more	Hydrocortisone, antivirals, tocizumab, sarilumab, immune- globulin therapy, anti-coagulation	~ 50 million USD
SOLIDARITY ^14^	14,200	7 4	52 Countries *	WHO and National Ministry of Health	Artesunate, infliximab, imatinib, remdesivir, hydroxychloroquine, lopinavir, INF-beta-a1	Total unknown
RECOVERY ^13^	47,289	14 10	194 sites across the UK, Ghana, Indonesia, Nepal, South Africa, and Vietnam	NIHR, UKRI, Wellcome	Corticosteroids, empagliflozin, sotrovimab, molnupiravir	~2.8 million USD
PRINCIPLE ^44^	9,724	7 4	UK	NIHR UKRI	Favipiravir, ivermectin, inhaled budesonide, azithromycin, doxycycline, colchicine,	~2.2 million USD
TOGETHER ^45^	6,000+	11 6	Brazil-Canada collaboration expanded to South Africa and Pakistan	Various philanthropic agencies, foundations grants	Hydroxychloroquine, Lopinavir/Rtonavir, Fluvoxamine, Ivermectin, Metformin, Doxazosin, Peginterferon Lambda, Fluvoxamine, Fluvoxamine + Molnupiravir, Fluvoxamine + Inhaled corticosteroid, placebo	NA
UPMC OPTIMISE-C19 ^46^	30,000	4 -	USA	University of Pittsburgh Medical Center	Monoclonal antibodies: Lilly Bamlanivimab,Regeneron Casirivimab + Imdevimab, Lilly Bamlanivimab + Etesevimab, Sotrovimab	NA
**Non-platform large trials**						
PANORAMIC ^47^	18,258	2 -	61 sites across UK	NIHR, UKRI, University of Oxford	Molnupiravir vs usual care	NA
COVID-OUT (factorial randomised trial) ^48^	1,000+	6 -	5 sites across USA	Various philanthropic agencies, foundations, grants	Metformin, ivermectin, fluvoxamine, or a combination of these medications versus placebo	NA

*For full list of countries, please refer to Appendix 4 NA Not Available

The RECOVERY trial revealed the dexamethasone findings in a July 2020 press release rather than waiting for official publication of results. This early press release – followed by detailed results in a preprint a few days later - resulted in an estimated 22,000 lives were saved ^41^. Approximately eight trial arms were added to RECOVERY and six arms were stopped early for futility since February 2020, reducing the need for multiple new or separate RCTs.

**Figure 8.**
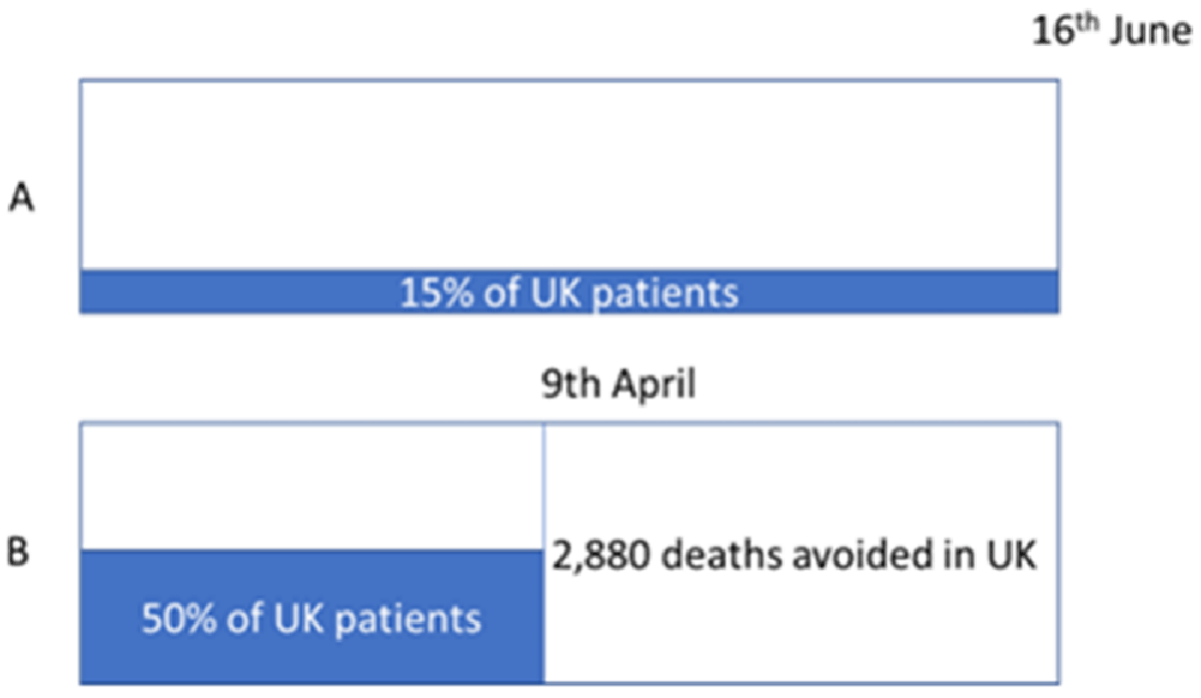
( **A**). The RECOVERY trial randomized around 15% of all hospitalized COVID-19 patients in the UK. ( **B**) If recruitment had been 50%, then it could have reported over 2 months earlier and saved an additional 2,880 lives.

RECOVERY was able to recruit rapidly in the first wave in the UK, and included 15% of all hospitalized patients. However, an interesting analysis ^42^ suggested that if 50% of patients had been recruited, then the early results (hydroxychloroquine and dexamethasone) could have been reported in mid- April rather than mid-June and saved an additional 2,880 lives in the UK alone ( Figure 8), and probably at least 10 times that globally. The same total number of patients would have been randomized, but the impact on others is greater. This suggests an ethical imperative to enable as many patients as possible to be randomized early.

***Discussion***

The total number of patients in the main platform trials in Table 4 is 135,000 patients randomized. This represents a substantial proportion of the patients in clinical trials. The living network meta- analysis project currently has publications for 463 studies which include 163,000 patients. The numbers are not directly comparable, as the platform trials are not all published. Nevertheless, it is indicative of their overall importance in the scheme of treatment evidence during the pandemic.

These platform trials have shown that the quick establishment of perpetual trials during a global pandemic is not only possible but essential. The amount of time, resources, and ultimately, patients’ lives saved thanks to these trials are quantifiable and significant. However, we need structural and regulatory changes that will enable platform trials to share essential trial details and the amassed data as readily, transparently, and widely as possible and widely as possible to reduce potential research waste due to doubling up similar studies, which could lead to failure to recruit sufficient participants, and undue discontinuation of study or its arms.”

## Q3. Models of good practice and lessons

**Q3. Are there models of good practice we can learn from? From past outbreaks, from COVID, more generally?**

**Key findings – Models of good practice and lessons**

•   Develop flexible mechanisms to expedite funding for high quality research on prioritised research questions.

•   Support pre-existing research networks to coordinate trial planning, design, conduct and practice change.

•   Across the spectrum of infectious disease research (prevention, transmission, treatment), prioritise large studies that use high quality designs.

•   Streamline set-up processes such as fast-track ethics and governance approvals.

•   Use simple and streamlined research processes to facilitate recruitment.

### Background

**Figure 9.**
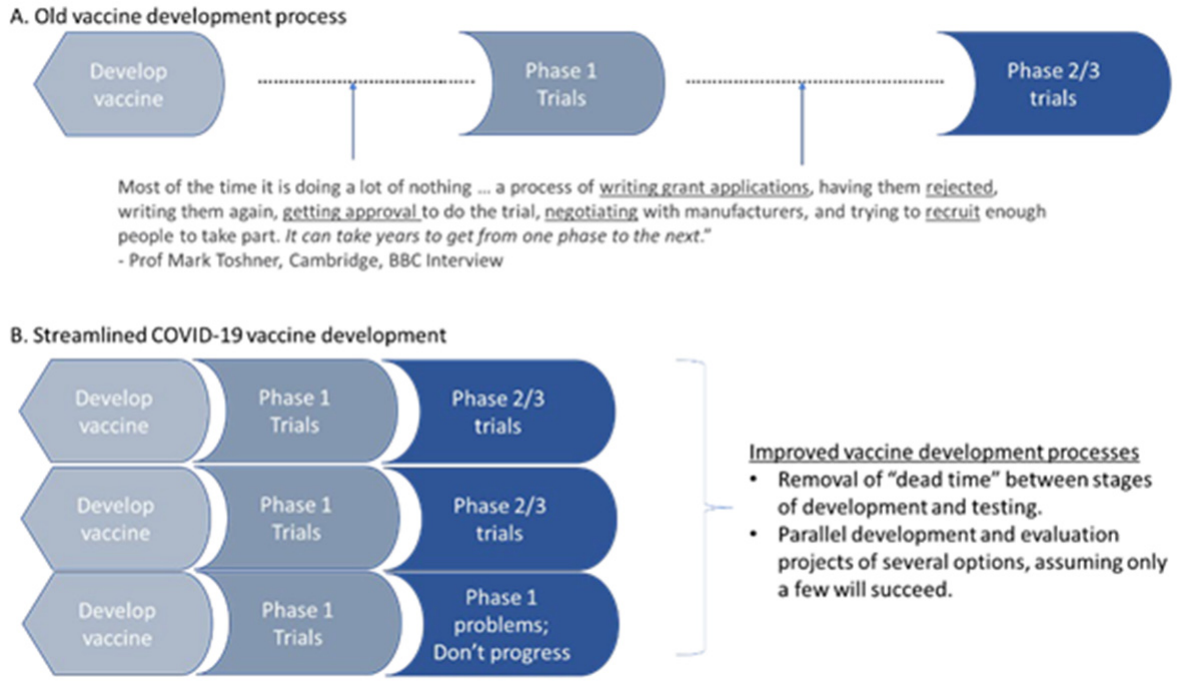
Compression of timeline for vaccine development from 10 to 1 year.

As previous sections have made clear, the rapid large scale adaptive trials have managed – for the first time during a pandemic – to provide rigorous evidence about the effectiveness of treatments in time to influence clinical usage. Several commentaries on these adaptive platform trials and general COVID-19 research ^49, 50^ have provided insights into factors that have hindered or facilitated success at differing stages of trial development, execution, and dissemination. For vaccines, many of the lessons were learned from failure to complete vaccine development in previous epidemics, such as Ebola, and led to the establishment of the Coalition for Epidemic Preparedness Innovations (CEPI). A major lesson had been better management of the vaccine development process (see Figure 9).

We aimed to compile some of the key lessons from platform trials successes and failures.

## Method

We conducted a literature search for commentaries on the major platform trials and COVID-19 trials more generally.

### Search strategy

Eight key articles (seed studies, e.g., 49– 51) were identified by an initial search by IEBH and WHO as high-quality commentaries on platform trials and COVID-19 research more broadly. We supplemented this with a citation search both backwards (checking reference lists) and forwards (identifying articles that cite our seed studies) using the SpiderCite tool in the Systematic Review Accelerator. The full list of all eight seed studies identified in the initial search is available in the appendices (Appendix 1). The supplementary citation search returned 314 potentially relevant studies.

### Data synthesis

To extract important lessons learned from models of research practice (both good and poor), we qualitatively synthesised the data across commentaries and categorised these as lessons for *Funders and Regulators*, or *Researchers and Research Networks*; and recommendations for different trial stages. These were mapped to stages of research development (from pre-design considerations through to evidence uptake). Two independent reviewers coded three randomly selected commentaries to develop the initial coding frame. A further two commentaries were also independently coded to check for consistency and refine the coding framework.

## Results

We found 322 articles in the COVID commentary search and a further 56 (N=378) in the search of key platform trial authors. We included 27 articles. Data saturation was reached (no new lessons were extracted) after 11 commentaries were coded.

**Figure 10.**
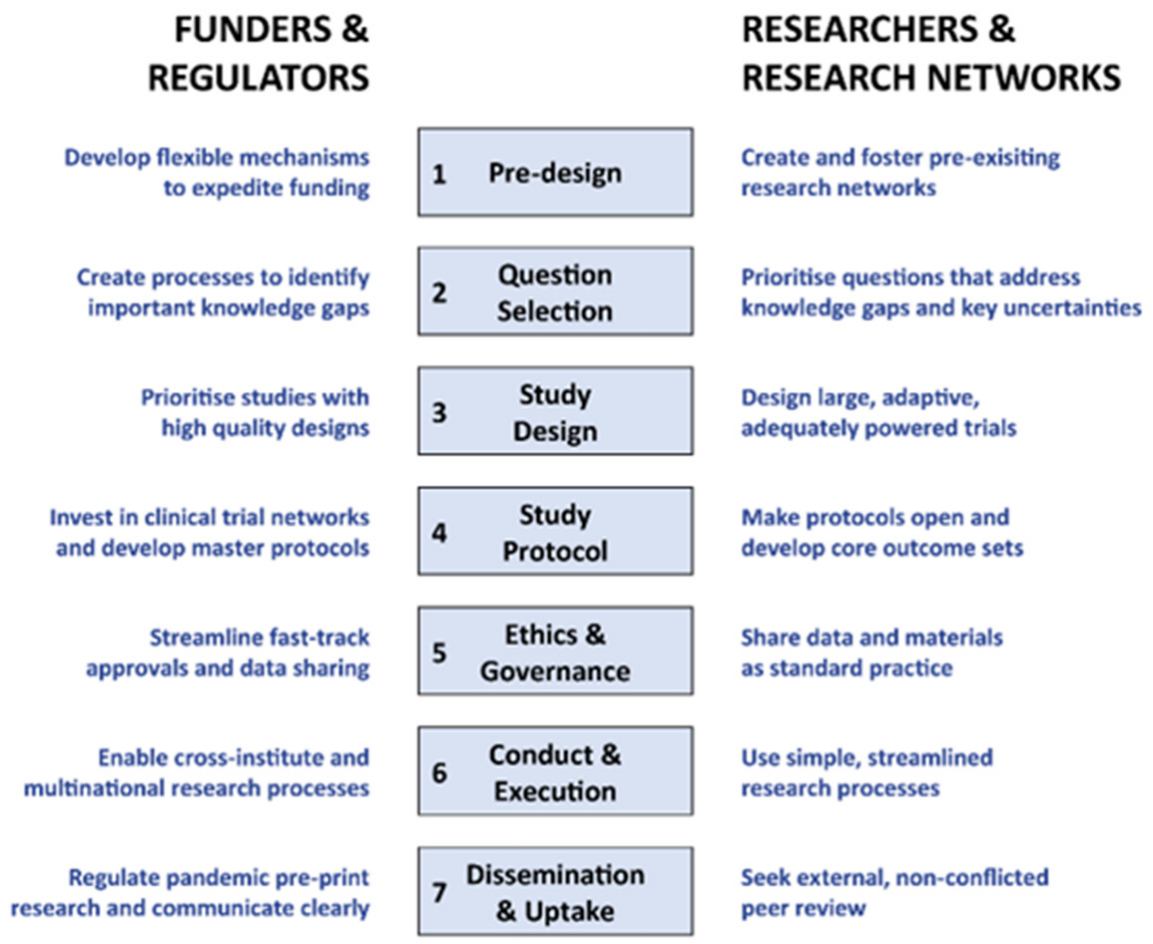
Key lessons learned for funders and regulators and researchers and their networks.

The synthesised lessons align to seven stages of research development: pre-design, question selection, study design, study protocol development, ethics and governance, study conduct and execution, and dissemination and uptake (see Figure 10).

### Pre-design

*“… large, simple, core studies should be national priorities, with coordinated support from chief medical officers, healthcare providers, funders and regulators to expedite set-up processes and promote rapid recruitment.”* - Tikkinen *et al.*, 2020 ^50^ Lead of Finnish SOLIDARITY arm

***Lessons for Funders and Regulators***

Develop flexible mechanisms to expedite funding for high quality research on prioritised research questions.

Speed and flexibility of funding were common themes across several commentaries. For example, Goossens *et al.* ^51^ suggest that a “ *mechanism should be in place to rapidly leverage [central] funding and to connect this funding with national public funding programmes. These funds provided by the [central funder] should incentivise academic and non-academic hospitals to participate in EU- funded clinical trials*”. In addition, Park *et al.* ^49^ recommend “ *Smarter investments for clinical trial research—whereby funds are allocated to clinical trials that are asking important research questions and that are well designed—should be made so that the funded trials have a high probability of generating conclusive evidence that can inform clinical practice and public health policies*.”

It is expected that funding mechanisms that prioritised important research questions would then provide “ *Safeguards to ensure public health relevance, independence and scientific excellence*.” ^52^

In addition to aligning research funding to prioritised research questions, expedited funding is critical to research success. Authors from the European Response Group reported “ *bottom-up funding mechanisms based on competitive calls are too slow*” and recommended there also needs to be “ *A top-down decision mechanism established at [a central] level*” would expedite the funding of priority questions ^52^. Similarly, Goossens *et al*. ^51^. suggested to “ *[d]evelop a mechanism to rapidly leverage pandemic funding and to connect [region] funding with national funding*.”

Finally, we note that most funding has been prioritised for vaccines and COVID treatments, with little to public health and social measures (PHSMs) that address preventing transmission. Glasziou and colleagues observed that “ *Considering the central importance of PHSMs for pandemic control, the uncertainties and controversies around their effects, and the immense research effort being put into vaccine and drug development, this lack of investment in public health measures is puzzling—at just 4% of global research funding for COVID-19*” ^53^.

***Lessons for researchers and research networks***

Support pre-existing research networks to coordinate trial planning, design, conduct and practice change.

Critically important for successful pandemic research were pre-existing research networks “ *EU and UK had established structures and procedures to facilitate a rapid, large-scale clinical research response in the event of a pandemic*” ^51^. In successful trials, these networks have enabled “ *Multi- country involvement [which has] allowed recruitment to shift with disease incidence*” ^50^.

The ability of these networks to engage key stakeholders to prioritise research was a key element of success. Park *et al*. ^49^ reported, “ *National-level collaboration and buy-in from major stakeholders are important components of clinical trial research*.” Tikkinen *et al*. ^50^ observed that “ *a strong letter of support from the Chief Medical Officers [CMOs] of England, Wales, Scotland and Northern Ireland emphasised that [the platform trial RECOVERY] was to be seen as part of clinical care*” with the CMOs issuing a statement that “ *Use of treatments outside of a trial, where participation was possible, is a wasted opportunity to create information that will benefit others*.”

Successful COVID-19 research hinged on coordination and collaboration between networks, funders and health services. Park and colleagues ^49^ argued that “ *[i]mportant lessons from COVID-19 have illustrated the need for pre-existing resource-efficient trial sites and capacity*. And Tikkinen *et al*. ^50^ recommended that *“[c]ountries should support clinical-trial networks that can quickly activate and adapt to contribute to large, simple multi-center trials that can study both older drugs and … new drugs….”*

In a pandemic, when coordination was absent, research waste was prolific. At the researcher level, “ *[t]he proliferation of studies that are largely duplicative is impactful [because it increases the likelihood of spurious findings] which could lead to further public confusion (or conviction) about the effectiveness of a therapy …. [and it] waste[s] financial and human resources at a time when healthcare and research resources are limited.”* ^54^. When there was a lack of coordination at the network level, confusion and research waste presided, *“[t]he health research funders of GloPID-R and WHO met … to discuss calls for proposals. Unfortunately, research priorities, processes to publish calls and select proposals, and procedural requirements were not sufficiently aligned*.” ^51^

### Question selection

*“efforts should be made to align research questions of critical and international importance*, … *This research should not only include clinical trials with therapeutics, but also basic and translational research on the natural history of the new disease, clinical course, transmission, risk factors, and more, facilitated by the clinical trial infrastructure*. *” -* Goosens *et al.*, 2021 ^51^. (REMAP-CAP and RECOVERY investigators).

***Lessons for funders and regulators***

Create rapid prioritisation processes to fund research questions that address important gaps in knowledge.

Broad statements within the lessons reflect the need to prioritise *“trials that evaluate potential treatment, prevention, or amelioration of COVID-19…”* ^54^ and that *“pandemic preparedness should be focused on protection over restriction”* ^55^. More specifically, Goossens *et al*. ^51^ noted that in response to the 2009 H1N1 influenza pandemic, “[the] *NIHR established the concept of Urgent Public Health (UPH) Research. To ensure the best use of NHS resources for clinical research during the acute phase of the pandemic, NIHR established a single UK-wide process to prioritise COVID-19 research as UPH research.*”

The European Response Group ^52^ identified the success of UK conducted trials such as RECOVERY was partly because of this planning; “ *The National Institute for Health Research established a single UK wide process to prioritize COVID-19 research as Urgent Public Health Research early in the pandemic”.* This foresight enabled fast-track reviews and single application submission with approval within days. Goossens ^51^ recommended that *“[a central] pandemic clinical research authority should be created to oversee pandemic preparation, clinical research response, and to prioritise clinical studies*.”.

Norton *et al.* *^56^* observed that although global research priorities were identified by the WHO, GloPID-R, and the UN, “ *other regions such as Latin America, one of the hardest hit by COVID-19, does not have a regional research agenda yet, and national research funding has not been prioritised by governments in recent decades.”* Without clarity and guidance, Angus ^57^ maintained that “ *Funding agencies are articulating varying views on priorities and processes*” resulting in “ *[h]undreds of COVID-19 trials have been registered on ClinicalTrials.gov, intending to test a wide array of interventions [and] [c]linicians and hospitals are bombarded with requests to participate.”*

***Lessons for researchers and research networks***

Prioritise research questions that address key uncertainties and gaps in pandemic knowledge.

Despite excellent hospital-based trials, clear gaps in covid19 knowledge remain. For example, Park and colleagues ^49^ identified that “[t] *he majority of trials have involved patients who have been admitted to hospital, and few clinical trials have investigated earlier stages of the disease process such as pre-exposure, or post-exposure and outpatient treatment*. Within these trials, we are still uncertain about correct dosage of some medications “... *study dose regimen comparisons have largely been absent in the current trial landscape of COVID-19. Failing to explore an adequate dose range or not including dosing that accounts for pharmacokinetic and pharmacodynamic variability in different patient populations can lead to an effective treatment being determined as falsely ineffective* ^49^.

**Figure 11.**
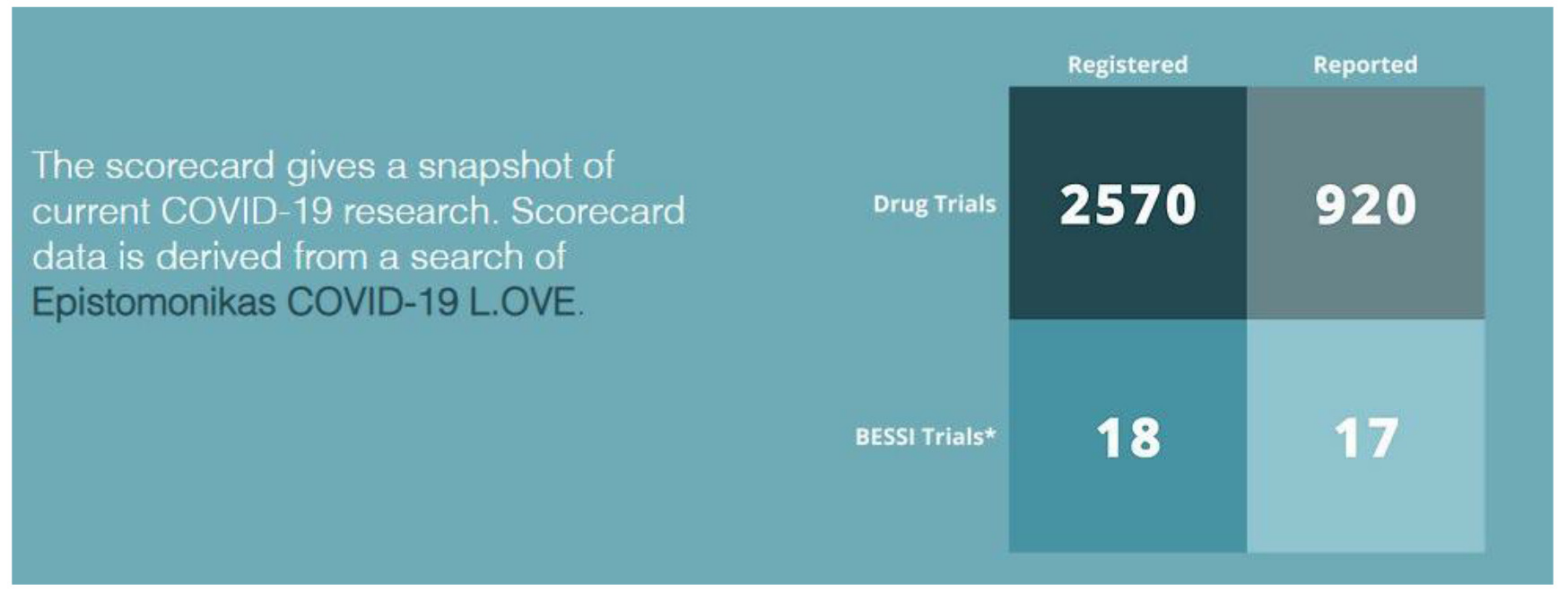
The BESSI Collaboration’s “scorecard” comparing the number of drug trials versus trials for Public Health and Social Measures (or BESSI – Behavioural, Environmental, Social & Systems Interventions), from https://www.bessi-collab.net/ (01/04/2022) (Reproduced with permission from the BESSI website producers).

Arguably, the largest gap in our pandemic knowledge is in public health and social measures. Despite thousands of trials of drug treatments, “ *much less has been done to evaluate the effects of public health and social measures (PHSMs) also known as non-pharmaceutical interventions (NPIs) or behavioural, environmental, social, and systems interventions (BESSIs) ^53^ – Figure 11.* Glasziou and colleagues *^53^* suggested a lesson to be learned was this paucity “ *The paucity of published observational or experimental studies on ventilation is one of the research tragedies of the pandemic.” ^53^*

Duplication of research questions (of both low and high priority) and subsequent waste in research efforts are lessons learned from poor models of practice. Park *et al.*reported, “ *With regard to treatments, although there are more than 100 unique therapeutic agents being investigated, there is also substantial overlap and duplicated trial efforts …”* ^49^.

### Study design

*“… rapid progress depends upon research that is rigorous, of scientific and societal value, and executed at the highest standards of scientific validity, including blinding to treatment assignment, randomization, and controls.” –* Bierer *et al.*, 2020 ^54^.

***Lessons for funders and regulators***

Across the spectrum of infectious disease research (prevention, transmission, treatment), prioritise large studies that use high quality designs.

To provide definitive and persuasive answers to clinical and public health questions, research needs to be high quality, well powered, and replicable. Obtaining clear and rigorous results provides a sound basis for guidelines, and also for subsequent research that builds on previous results, for example, RECOVERY provided a clear result for dexamethasone, allowing for subsequent trials looking at the impact of different doses. “ *Perpetual clinical trials provide an opportunity to answer multiple questions about several interventions in the most efficient way imagined, paving a pathway for continuous improvement*.” ^49^. COVID-19 platform trials with multi-country and large samples provided clear answers to prioritised treatment questions (see Q1 and Q2). Predefined platform trials have been described as “ *an efficient approach to knowledge acquisition*” ^54^ and have the capacity to compare multiple arms simultaneously. For example, “ *the World Health Organization planned the platform trial SOLIDARITY, a trial that directly compares four treatments for COVID-19 (remdesivir, chloroquine and hydroxychloroquine, lopinavir-ritonavir, and lopinavir-ritonavir plus interferon- beta)*.” ^54^

The consequence of not prioritising and funding high quality, coordinated research designs results in *“[i]nconclusive research findings from many clinical trials*” ^49^ or “ *dangerous conclusions”* ^58^ particularly when unvetted research is quickly uploaded to pre-publication sites and remain *“available to researchers, with no mention within the article of the existence of the post-publication reviews as a warning.”* ^58^

There are ethical issues for funders and regulators who fail to promote high quality research. Park and colleagues ^49^ reported “ *The preponderance of two-arm trials also leads to other important issues. Instead of doing multi-arm or platform trials with a common control group, the prevalence of two-arm trials has resulted in multiple redundant control groups.”* Worse, *“[m]ost studies were observational in design, exposing thousands of patients with COVID-19 to compassionate use drugs without high-quality data collection*.” ^51^

***Lessons for Researchers and Research Networks***

Improve methodological rigour and design large, adaptive, adequately powered trials.

Much of the research about COVID-19 is low quality. Members of a consortium including GloPID-R, UKCDR, and COVID-19 Clinical Research Coalition observed that *“[l]arge international trials, including RECOVERY, REMAP-CAP, and WHO SOLIDARITY, have provided definitive answers for the treatment of hospitalised COVID-19 patients. …[However,] [t]hinly spread global funding has, in other instances, resulted in a proliferation of underpowered, heterogeneous studies that have had little impact.”* ^56^

Park observed ^49^ “ *an overwhelming number of COVID-19 clinical trials … are being done without methodological rigour and adequate planning*.” Where *“[t]rials have, on average, planned sample sizes of fewer than 100 participants, and are typically evaluating only one experimental intervention”* ^49^. Most trials are argued to have been so underpowered that they “ *will not provide sufficient statistical power to detect a meaningful treatment effect….[and] [m]ost will never achieve their target recruitment numbers* ^49^. It has been argued that many trials were inappropriately designed to “ *demonstrate large, unrealistic treatment benefits, in order to justify sample sizes of several hundreds of patients per study group*” ^51^.

These observations extend to the small number of studies of public health and social measures where “ *the quality of the current evidence would be graded….as low or very low, as it consists of mainly observational studies with poor methods (biases in measurement of outcomes, classification of PHSM, and missing data), and high heterogeneity of effect size* ^53^. Therefore, it has been suggested that the “ *findings from the observational studies might be better interpreted as the impact of a bundle of correlated protective behaviours for which the individual behaviours are a marker”.* ^53^.

Some research areas did very well in delivering answers and benefits in record time aspects and in the most challenging circumstances. However, there is also a clear need for improvement in some areas. Poor research designs with inadequate power are both wasteful and negligent. These lessons of poor practice models exacerbate trial duplicity of low priority studies and compete for scarce resources. Bierer and colleagues ^54^ reported that *“[a] multiplicity of open trials at an institution … risks the possibility that no trial will complete enrolment, that the number of patients in any institution will simply not support the successful execution of all studies. These considerations strongly support minimizing the number of currently enrolling studies at the same institution, especially when similar studies evaluating the same agent or intervention are undertaken elsewhere.”*

### Study protocol

*“Establishing a master protocol for a platform trial can help establish an efficient research ecosystem that is prepared for a future pandemic–” -* Park *et al.*, 2021 ^49^.

***Lessons for funders and Regulators***

Work with clinical trial networks to develop master protocols with adaptive designs.

“ *In previous pandemics, large-scale randomised trials were generally not set up in time*.” ^50^. Goossens and colleagues ^51^ argued that efficient coordination and collaboration would “.. *be more effectively facilitated by investing globally connected clinical trial networks, structured through platform trials, and established under a master protocol framework*.”. The successful platform trials adapted for COVID-19 (e.g., RECOVERY, SOLIDARITY) benefitted from pre-existing networks and pre- existing adaptable core pandemic protocols. “ *The COVID-19 pandemic has catalysed the acceptance of master protocols by the research community as there is a clear need for more structured and sustainable approaches to clinical trial evaluation* ^49^.

Master protocols of perpetual clinical trials with “ *predefined triggers for efficacy or futility at planned adaptive analyses*” ^51^ “ *provide an opportunity to answer multiple questions about several interventions in the most efficient way imagined, paving a pathway for continuous improvement*” ^49^. Adaptive master protocols such as REMAP-CAP had a pre-written appendix in the original master protocol to include influenza-like patients if a pandemic occurred. Park ^49^ suggested that this adaptive design “ *could represent an approach that funders and future trialists should consider by having a worst-case scenario in their planned master protocols”.*

By developing and using master protocols, they “… *can be leveraged to encourage collaborations to generate scientific evidence in a timely manner while promoting rigorous standards between different regions of the world*” ^49^ and are a “ *far more efficient [way] to use designs that leverage a common platform for trial entry, data collection, and testing of multiple therapies*.” ^57^.

***Lessons for researchers and research networks***

Develop core outcome sets and register protocols.

Despite the volume of COVID-19 research, very little can be systematically synthesised. For hospital trials, Park ^49^) observed “[ *f]or COVID-19, there will be many challenges of doing meta-analyses with aggregated reported data. First, even within trials studying the same intervention, there is substantial heterogeneity in dose, duration, endpoints, and data collected between different trials*”.

The situation is somewhat worse for trials of public health and social measures where *“meta- analysis was not possible for the outcomes of quarantine and isolation, universal lockdowns, and closures of borders, schools, and workplaces*” ^53^.

As Bierer and colleagues reported ^54^ “... *global collaboration is necessary to enable insights learned in one location to be applied to the next and to build upon knowledge, not reinvent it. For this to occur, common vocabularies and means of recording symptoms, co-morbidities, demographic and non-demographic characteristics of the individuals and agreement on common, objective endpoints and their definitions;...must be in place, all of which may then be applied to rigorous research methodologies in the service of public health*.” Bierer also noted that efforts “ *to develop common data standards to enable data interoperability will ultimately save time and resources*” ^54^. Development of these common data standards would require the collaboration of multiple stakeholders including evidence synthesis researchers, guideline developers, and agencies such as the HTA.

Some commentators strongly advocated that researchers register study protocols, as a way of reducing research waste. Besancon ^58^ suggested “ *Both pre-registration and registered reports contribute to a better visibility of ongoing research and should be used at institution levels to coordinate research projects at an international level in a more efficient way, in order to optimise resources.*” Besancon ^58^ also noted that registration holds researchers to account to show that the “ *published study has been conducted and analysed as planned, thus limiting the risks of changes to the design, methods or outcomes in response to the data obtained other than flexibility allowed by the protocol (in case of interim analyses of adaptive designs).*

There are multiple platforms to register research protocols. For example, PROSPERO for systematic reviews, ClinicalTrials.gov for trials, or the Open Science Framework for all study designs. Bierer *et al.* ^54^ recommended “ *study preregistration on dedicated platforms (e.g., ClinicalTrials.gov, OSF, or AsPredicted), with a thorough description of the study design, ethical approval, methods for data collection and data analysis”* To ensure speedy review of protocols Besancon *et al*., ^58^ observed “ *some platforms for the submission of registered reports put in place measures to guarantee a timely review of COVID-19 protocols: stage 1 review of registered reports at Royal Society Open Science are per formed within 7 days*.”

### Ethics and governance

*“..established researchers should encourage a transition to transparent research; institutions and funding agencies should diversify research evaluations; journals, editorial boards, and funding agencies should make all Open Science practices the de facto standard for submissions.”*

- Besancon *et al.*, ^58^

***Lessons for funders and regulators***

Streamline set-up processes such as fast-track ethics and governance approvals.

Streamlining the start-up processes of research is critical. A significant impediment to improving pandemic research infrastructure is the lack of data sharing, including across trials. Park *et al*. ^49^ reported that the “inadequate *number of data sharing mechanisms that exists for COVID-19 is a major obstacle*” with *“no coordinated global approach to aggregate data”* ^49^. Funders and regulators have an opportunity to improve trial data useability by mandating “ *the widespread implementation of Open Science principles – known to increase the rigour, reliability and reproducibility of scientific results – [which] could help optimize research [and] improve health outcomes and economic costs* ^58^. Goossens ^51^ recommended leading regional authorities “ *should develop models and procedures to mandate data centralisation and sharing*.”

Potential solutions already exist for these mechanisms: Besancon *et al.* ^58^ observed several, including noting that it was “ *already mandatory for some types of clinical trials, and registered reports should be used more systematically*” therefore extending these mandates would not be onerous and existing initiatives such as “ *OpenSAFELY, have emerged to make data available to researchers while complying with the legislation regulating the use of medical data*”. Coordinating and regulating Open Science principles and data sharing specifically would improve efficient knowledge gains from pandemic research.

**Figure 12.**
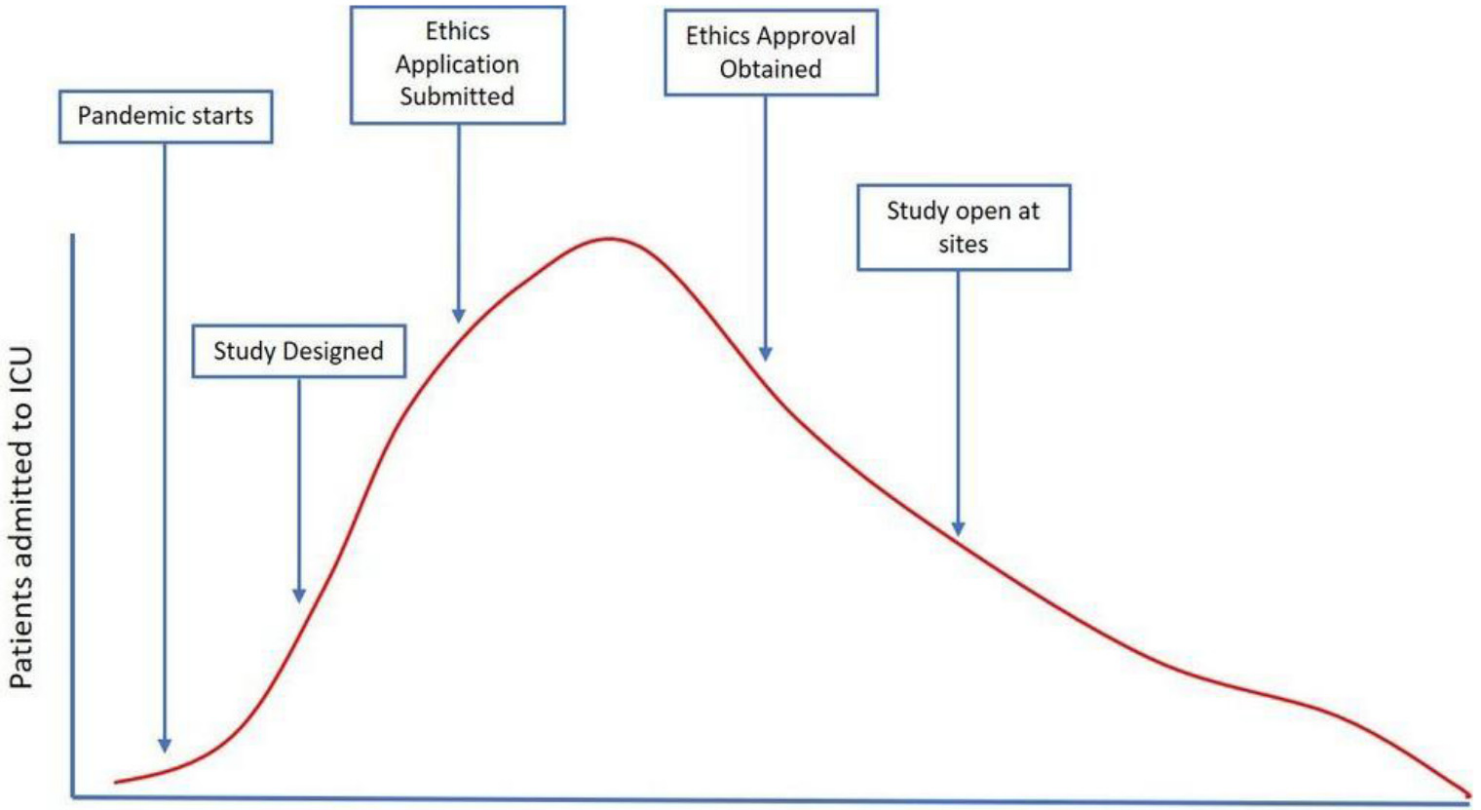
The time taken to design, gain approvals, and implement a research trial may mean that a large proportion of the first wave recruitment is missed (Figure taken from https://www.remapcap.org/pandemic-preparedness; Reproduced with permission from the website managers).

Approval processes across settings were disjointed, with significant differences between protocol/ethic approval times across regions. For REMAP-CAP, protocol approval at European sites ranged between “ *7 days to over 12 months*” ^51^, despite REMAP-CAPs clearly recognizing the rapid processes needed (see Figure 12). One of the difficulties in approval for REMAP-CAP across sites was that “ *each individual site needed to be contracted by the study sponsor*”, therefore markedly impeding fast-track approval. These *“[p]rolonged evaluation times are therefore obstacles … to the subsequent rapid development of best clinical care for patients*” ^52^. Tikkinen ^50^ also reported that trials “ *were delayed by approvals from national drug regulators, ethics committees or health ministries and missed the first wave’s peak*”.

Of note, the EU Response Group suggested that regulations introduced in January 2022 “ *will ensure that rules for conducting clinical trials are identical throughout the European Union (EU) and will also allow a coordinated assessment of clinical trial applications and especially the protocol and the product between Member States*”. It would be prudent to monitor the consequences of this regulation, and if successful, consider adopting and adapting similar strategies for other regions.

Once broad approval of trials is achieved, local variations and *“[a]mendments must be subject to fast-track review”* ^52^.

***Lessons for Researchers and Research Networks***

Share data and codes as standard practice.

Although a lesson for funders and regulators was to mandate data sharing, a similar lesson for researchers and their networks was to share data as part of standard research practice. As Besancon and colleagues ^58^ wrote, *“[d]ata should be shared by default: authors should not be able to submit a manuscript if they do not provide access to raw data and analysis scripts or a valid reason why they think it is not feasible”.* Platforms to share data and codes exist. Besancon ^58^ reported *“[t]he use of source code sharing platforms, such as GitHub or open source alternatives such as GitLab, is becoming common and has even been advised to improve open science behaviour. The code should be published under a free license to encourage re-use and further developments, and when possible, open source software and programming languages should be preferred to maximise accessibility and reproducibility”*.

### Conduct and execution

*“We urgently need defined standards for trial execution during pandemics.”* - Goossens *et al.*, 2021 *^51^*.

***Lessons for funders and regulators***

Enable cross-institutional and multinational research processes.

A principle lesson for funders and regulators was to facilitate clear, coordinated pathways for local institutions to conduct large-scale trials. Park *et al.* ^49^ reported the coordinating function of WHO “ *streamlined the patient enrolment and centralised web-based randomisation procedures that do not require paperwork*”. This coordinated effort decreased the “ *burden of research duties in participating hospitals”*. They also provided a “ *centralised randomisation and data capture system, harmonised statistical support … with interim analyses being monitored by a global data and safety monitoring committee …”* ^49^.

Funders and regulators could also facilitate research through promotion and incentives. Goossens and colleagues ^51^ observed that although RECAP-MAP was designed and commenced prior to the COVID-19 pandemic, “ *there was a low sense of urgency for pandemic preparedness and the financial reimbursement in this EU funded project was hardly competitive to the many other study opportunities in intensive care units.”*

***Lessons for researchers and research networks***

Use simple and streamlined research processes to facilitate recruitment.

The more simplified the research procedures were, the easier it was to facilitate research and reduce the burden on clinicians. RECOVERY is an exemplar. First, to participate in the RECOVERY trial, *“[a] standard contract was issued to sites with a “take it or leave it” approach, allowing no room for local negotiation or adaptation* ^51^. This saved much time. Second, *“[RECOVERY] sought to achieve reliability and quality by design rather than by compliance with good clinical practice or site monitors, relying instead on centralised computer checks on site behaviour and patient compliance, and utilising central … medical records of treatment and outcome.”* ^50^. Finally, the eligibility criteria were simple. For example, patient written consent was waived “ *where the medical emergency rendered this inappropriate*.” ^50^ These simple, streamlined processes enabled easy participation in this trial so that “ *many of the less-research-experienced hospitals [became] among the best recruiters.*” ^50^

### Dissemination and uptake

*“[H]igh quality dissemination of scientific information is essential to an appropriate public health response to a crisis such as COVID-19” -* Besancon *et al.*, 2021 ^58^.

***Lessons for funders and regulators***

Regulate pandemic pre-print research and foster clear, accurate, and trustworthy communication of key findings.

Some systems have been improved to balance the need for rapidly acquired, yet accurate, knowledge. For example, some “ *major publishers …] have made newly written COVID-19 related articles freely accessible to all (Open Access)*” and “ *a number of journals have recently implemented specific policies to fast-track COVID-19-related research*” ^58^. However, the proliferation of unvetted COVID-19 pre-print research has led to the dissemination and use of knowledge from poor quality studies. Conflicts of interest were also exacerbated – for example, “ *Among the 699 articles accepted within a day, an editorial conflict of interest was observed in 297 (42.5%) articles*” ^58^. *“Fast- tracking of peer-review should therefore only be done when scientific rigour can be maintained as its loss might lead to disastrous consequences for public health as a whole.”* ^58^

With the immense amount of COVID-19 research information available, clear, accurate, and trustworthy dissemination of it is critical. “ *Only through global communication and collaboration will common approaches be adopted and insights advanced.”* ^54^ Both good and poor examples of research communication occurred. Tikkinen *et al.* ^50^ reported that “ *coordinated support from chief medical officers, healthcare providers, funders and regulators to expedite set-up processes and promote rapid recruitment”* was a key success of RECOVERY in gaining public and clinician trust.

Trust in key stakeholders who disseminated research information through policy was echoed by Williams and colleagues ^55^ and recommended fostering trust as a “ *long term investment for the next pandemic”.* There is a risk in publicising research that has not been peer-reviewed and challenges in communicating research to journalists and publics keen for information.

***Lessons for researchers and research networks***

Seek external, non-conflicted peer review.

Pre-prints, reviews, and journal access can be regulated but the onus is also on researchers to be judicious in their quest for knowledge. As Besancon ^58^ opined “[while] *the faster embracing of Open Science during the pandemic is a step towards more accessible and transparent research, we also express concerns about the adoption of these practices for early and non-validated findings*.” The “ *floods of preprints and publications from COVID-19 research have created confusion, not only among the scientific community, but also among the public, who are eagerly waiting for the scientific community*.” ^49^. Review and editorial shortcuts *jeopardis[ed] the integrity of the editorial process and putting the rigour of scientific publications at risk”* ^58^ and on occassion, led to “ *papers, later retracted, … informing public health policy*”. The more confusion in research, the less trust.

## Q4. Potential conflicts and points of tension

**Q4. What are some of the potential conflicts we need to avoid?**

**Key findings – Potential conflicts and points of tension**

•   When considering the conflict between clinical practice and trials, the need to act must be balanced with the need to know

•   In academic versus public health interests, there are tensions between publication and funding driven indicators and the public good

•   Between the commercial and non-commercial sectors there are frictions with paywalls, profits and hype

•   Allocating scarce resources between healthcare and research is challenging

•   When treating patients through trials, balancing individual and collective rights must be carefully considered.

### Methods

In parallel with the search for “lessons” (see Methods in previous section), potential conflicts were identified in the included commentaries and deductively coded to reflect the possible tensions surrounding four key intersections:

1. Clinical care and pandemic trials;
2. Academic interests that drive small trials rather than contributing to larger trials;
3. Commercial and non-commercial interests; and
4. The distribution of scarce resources during an emergency.

We also iteratively developed themes reflective of potential conflicts that were not captured by the four key themes above.

Four key areas were identified and summarised as in the categories: clinical practice vs research trials; academic vs public health interest; commercial vs non-commercial interests; resources for healthcare vs resources for research. We also identified some ethical tensions surrounding the conduct of, and participation in, research trials.

### Results

***Clinical practice vs research trials***

*“[H]ow does the fiduciary relationship between a patient and their doctor modify the boundaries between research and care” -* Bierer *et al.*, 2020 ^54^.

Early in the pandemic, Angus ^57^ wrote about the tension between exploiting knowledge that was already known compared with exploring unknowns. He described this as a tension between clinicians and researchers. He wrote *“[t]he world is united regarding the goal of ending the coronavirus disease 2019 (COVID-19) pandemic but not the strategy to achieve that goal. One stark example is the debate over whether to prescribe available therapies, …. or test these drugs in randomized clinical trials*” ^57^. He goes on to discuss the dilemma experienced by the clinician where he portends, *“[r]andomization is profoundly uncomfortable*..” as opposed to guidelines or standard practice where clinicians might believe “ *that the chance of benefit outweighs the chance of harm*”. However, Angus ^57^ also reflected that “ *… the benefits of accelerated learning through participation in the trial, as well as the consequences of delayed knowledge generation through failure to participate, feel abstract, remote, difficult to calibrate, and beyond the physician’s responsibility”* ^57^.

In an opposing view, Bierer *et al.* ^54^ observed *“one major unfortunate consequence of facilitating off-label use, [and not participating in trials] …, is that future patients will not benefit from reliable evidence as to the efficacy and safety of [drug name] or off-label treatment with other, already approved and marketed agents.”* ^54^. Although, Tikkinen and colleagues ^50^ concluded, that “ *several false claims of efficacy have emerged from non-randomized comparisons (often misleadingly referred to as ‘real world evidence’), and it has been refreshing to see how …[these] claims..”* can be refuted by “ *large scale randomized trials*”, how to improve clinician uptake of large-scale trials and reduce the tension between ‘exploiting knowns’ and ‘exploring unknowns’ will be a critical tension to resolve.

***Academic vs public health interests***

*“The new norm of publishing: quantity over quality” -* Park *et al.*, 2021 ^49^.

Several commentators observed the conflict surrounding the academic pursuit of publications, funding and reputation and the interests of public health. In a pre-COVID-19 pandemic publication, Kieny ^59^ put it bluntly, *“[the west African Ebola epidemic] showed the competitive spirit that is typical of scientific research, where “getting there first” is not just a matter of professional pride but also carries financial risks and the potential for large profits*” ^59^. Also exemplifying this tension, Angus ^57^ wrote, *“[e]verywhere, those who would design and conduct trials are competing for funds and priority review.”* ^57^

The excess of pre-print repositories exacerbated this tension because authors could “count” these studies without peer review and formal publication. This proliferation of low-quality research is illustrated in earlier results. Prompting the EU Response Group (REF) to call for “ *protocol pre- submission review*” ^52^. Besancon and co-authors ^58^ found that we have found that “ *the fast- tracking of peer-reviews on COVID-19 manuscripts, which was needed to give vital treatment directives to health authorities as quickly as possible, led to potentially suspicious peer-reviewing times often combined with editorial conflicts of interest and a lack of transparency of the reviewing process.*” ^58^

Park *et al.*, ^49^ appealed for “ *the need to share and collaborate openly supersedes personal careers or organisational goals*” ^49^ and observed we should “ *aim is to strike a balance between quickly disseminating data via preprint servers while ensuring that the work is scientifically credible*” ^49^.

***Commercial vs non-commercial interests***

*“[a challenge for REMAP-CAP recruitment was competing with] media attention and political support for some treatment options, such as hydroxychloroquine and lopinavir–ritonavir, and obstructing randomisation options in many countries.” -* Goossens *et al.*, ^51^

Commercial interests (either journal, media, or pharmaceutical) were raised as potential conflicts. For example, some peer-reviewed research was behind journal paywalls ^58^, which benefitted the journals but not public health more broadly. Further reputational damage to journals through retraction of articles is a potential conflict where some journals chose to “ *not to withdraw the publication, but instead encourage the submission of comments, will help increase the impact of the journal, despite the poor quality of the original publication.”* ^58^

Finally, Besancon and colleagues ^58^ also raised possible conflicts (and queried the ethics) of some pharmaceutical trials, “ *Among possible ethical risks, [another author] identified over-recruitment in trials, the conduct of human vaccine studies before the completion of animal studies, and the neglect of adverse effects in drugs studies. An example of the last is the little consideration given to the known cardiotoxicity of the combination of hydroxychloroquine and azithromycin early on in the pandemic”* ^58^. Tension between commercial interests were also reported by Angus ^57^, *“[p]harmaceutical trials are moving quickly, but in competition with each other”* ^57^.

***Resources for healthcare vs resources for research***

*“[division of labour between clinical practice and clinical research]… [have huge costs]… including delays in knowledge acquisition and dissemination. In normal times, these costs are somewhat suppressed or ignored, but in a crisis such as the COVID-19 pandemic, they come into sharp focus.”*

- Angus *et al.*, 2020 ^57^

Allocation of scarce resources to clinical care and/or research was a conflict raised by the most commentators. Allocation of human resources in health emergencies was raised by Park *et al.* ^49^ in that the *“[l]ong-term human resource utilisation should be viewed as a top priority, as it can be an effective measure to address common concerns regarding education or training and the capacity of the region to undertake high-quality clinical trial research”* ^49^. But also the conflict between funding research and funding public health initiatives, *“[g]iven the scarcity of funding, funding clinical trial research can mean that there is less funding available to implement public health initiatives(and vice versa)”* ^49^.

Duplication of trials, for example those “ *being run in the same region or institution will ultimately compete for participants and delay recruitment into well designed trials that can provide reliable scientific evidence*” ^49^. They also “…. *waste financial and human resources at a time when healthcare and research resources are limited.[…] resources used by duplicative trials may require delaying or foreclosing the initiation of trials that propose to study novel treatments*.” ^54^ Given the extent of primary research and the questionable quality of some primary research, prioritising and funding high quality evidence synthesis research that can be expertly and quickly updated is critical in future outbreaks.

***Patient choice vs trial protocol***

*“[S]ociety must balance the need for information and knowledge in the service of public health with the fiduciary responsibilities of the clinician to his or her patient and the patient’s right to consent or not, concordant with their personal wishes”* - Bierer *et al.*, 2020. ^54^

Bierer and colleagues ^54^ were the only commentary to identify the tension between the individual patient’s right to choose and the execution and conduct of a clinical trial. They queried if healthcare institutions and their investigators were committed to gathering evidence through RCTs, “ *is it ethically appropriate to restrict access to an otherwise approved and marketed, relatively safe, medication*?” ^54^ *“… [W]ould the doctor be duty-bound to inform the patient that the drug is available elsewhere by prescription?”*

The ethical conduct of the trial was also raised as a possible source of tension, particularly when they “ *begin, continue, halt and resume*” which create “ *an ongoing and evolving ethical challenge*.” ^54^.

Finally, they raised the necessity of the swift nature of trial evolution compared with the need to prioritise participant safety to ensure “ *risks could be mitigated and whether trial and data integrity could be assured*” ^54^.

## Data Availability

No data are associated with this article. Open Science Framework: Clinical trials and their impact on policy during COVID-19: a review, https://doi.org/10.17605/OSF.IO/4YCPZ ^60^. This project contains the following extended data: COVID trials Wellcome Appendices 1–4.docx Data are available under the terms of the Creative Commons Attribution 4.0 International license (CC-BY 4.0).
