# Supplementary material for: Clinical trials and their impact on policy during COVID-19: a review
Source: Wellcome Open Res. 2024 Jan 30;9:20. [Version 1] doi: 10.12688/wellcomeopenres.19305.1 (PMC10905118; doi:10.12688/wellcomeopenres.19305.1)
Supplement: Peer review history

# Reviewer report: Reviewer response for version 1

**DOI:** 10.21956/wellcomeopenres.21388.r74106

**Published:** 2024-02-28

**Authors:** Frank Hulstaert

**Affiliations:** 1 Belgian Health Care Knowledge Centre, Brussels, Belgium

The paper provides a nice overview of the strengths and weaknesses of the way the world managed COVID using platform trials, starting from the published scientific literature. The structure is clear with 4 research questions that are addressed separately. The comments are grouped accordingly.

- Q1. Social value in conducting trials and uptake into policy.

Some of the publications may be in the grey literature, e.g. EU HTA bodies (grouped under EUnetHTA) worked together to update regularly the clinical evidence supporting specific COVID treatments and diagnostics to inform the national decision makers https://www.eunethta.eu/covid-19-treatment/

 In most EU countries the monoclonals and antivirals were procured nationally without passing through the regular HTA and reimbursement procedure. A separate process to access these drugs may have been used and therefore these drugs may not be included in the standard administrative billing or prescription data. In the discussion on uptake of monoclonals it may be key to mention the issue of (in-vitro) resistance, sometimes quickly after the monoclonal(s) became available for use. As the rate of hospitalisation decreased based on vaccination and less pathogenic circulating variants, also the target population for antivirals may be changing.

 Using platform trials as a starting point, the paper focuses on dexamethasone and remdesivir, and in-hospital trials in general. It remains difficult to estimate the real overall effect of using dexamethasone. The effect of dexamethasone (and IL-6 blockers) on ICU mortality was seen mainly in settings with a rather high COVID ICU mortality of 30% (UK, NL) whereas the effect might not be significant in settings with already a low COVID ICU mortality rate like 13% (Norway, BE).

- Q2. Issues with adaptive trial designs.

The importance of a high participation rate (15%, but ideally higher) in platform clinical trials is mentioned in the context of RECOVERY. A very high participation rate of 70% has been seen for cardiovascular registry-based randomised trials in Sweden, so a high uptake is possible. It has been noticed by multiple clinical investigators in UK and Belgium that specific minority populations had a low participation rate in COVID clinical trials. After the base COVID vaccinations the COVID hospitalisations were seen more often in non-vaccinated individuals. A drop in trial participation rate was also observed, suggesting the same subjects refusing vaccination may also refuse trial participation. It has been suggested this might be related to a lack of trust in the public sector.

 It may be relevant to mention that Panoramic also studies Paxlovid, with recruitment planned until end of March 2024.

 For the table, maybe add why certain platform trials are considered key and some are not included eg the NIH ACTT-1,2,3,4 trials, some are adaptive platform trials.

- Q3. Models of good practice.

Platform trials have been set up most rapidly in single countries when pushed and funded by government and when infrastructure (and study nurses) was in place. For international trials to have this top down approach in EU there may be a need to create a European Institute for Health Research that can move quickly and make use of an existing trial infrastructure in the hospital as well as in the outpatient setting.

 Good funding practices deserve to be mentioned too, as this has an impact on trial completion rates. This includes a review of the relevance and quality of the trial, supported by professional statisticians and data managers, and patients, a check of the feasibility of the trial supported by an advance payment, funding that is sufficient to cover all necessary trial activities of the sponsor and the trial sites, and finally, a payment schedule that is milestone based with last payment made when trial results are publicly reported. [1]

 Another lesson learned for international platform trials is that template contractual clauses between trial funders, pharmaceutical companies, sponsor and sites should be discussed and agreed upon in advance as much as possible. Belgium did finally not participate to Solidarity for contract related reasons. There is no time at the start of a pandemic to have these discussions. As mentioned for RECOVERY, a take it or leave it approach can then be used.

 Another hurdle, seen for drugs used in-hospital like IL-6 inhibitors, was the stockpiling by hospitals during the early days of the pandemic, making it a challenge to timely purchase sufficient drug quantities for a clinical trial. [2]

 Under lessons for researchers and research networks, the relative lack of trials outside of the hospital setting is mentioned. One of the first papers on a possible mechanism to block viral entry pointed to the repurposing of TMPRSS2 inhibitors (camostat and nafamostat, marketed for many years in Japan and South-Korea) [3].

 However, it proved very difficult to obtain access to these existing drugs for inclusion in a trial early on during the pandemic. Companies were not interested, not willing to provide or even sell their drug. In absence of a company with expertise in drug development, there was no national or international public or academic entity ready to take over this important coordinating role for repurposed old drugs. This is probably why early phase pilot trials were repeated at different places and without real coordination and all lacking the necessary non-clinical PK/PD data support. [4].

 There is indeed a relative lack of platform trials in at risk patients evaluating drugs that aim to keep these patients out of the hospital. This was the case for monoclonals like sotrovimab or combinations like Evusheld. In addition, challenges included ever changing viral variants. Only in England the PANORAMIC trial recruited very well for molnupiravir and rather well for Paxlovid, heavily supported by government. In Belgium the Dawn antivirals platform trial did not recruit well. Reasons were the lack of an existing trial infrastructure in the primary care setting and the local quarantine measures hampering the trial consent process and study medication logistics. [5]In the early days the lack of reliable diagnostic tools in primary care also hampered research in this setting.

 In particular for international GP platform trials it seems key to standardise quarantine rules across countries as much as possible.

 A lesson for funders: the other ongoing (non-COVID) trials may need extra funding after having been interrupted by a pandemic.

 Ethics review must respect the Declaration of Helsinki, which stipulates trials should include an appropriate comparator and endpoint. How come so many COVID single arm and underpowered randomised trials got a positive opinion?

- Q4. Potential conflicts to avoid?

Resources for healthcare vs resources for research was found to be an issue especially for GP trials where GPs were already too busy with their clinical routine work. There is a need for dedicated personnel and infrastructure also in this setting as is the case in the UK.

 Typo in table 3: nirmatrevir.

Is the review written in accessible language?

Yes

Are all factual statements correct and adequately supported by citations?

Yes

Are the conclusions drawn appropriate in the context of the current research literature?

Partly

Is the topic of the review discussed comprehensively in the context of the current literature?

Partly

Reviewer Expertise:

Previously clinical development of medicinal products, currently HTA and funding of comparative effectiveness trials and repurposing trials. Coordinated academic non commercial randomised trials for COVID in Belgium.

I confirm that I have read this submission and believe that I have an appropriate level of expertise to confirm that it is of an acceptable scientific standard.

## References

[1] : Budgeting of non-commercial clinical trials: development of a budget tool by a public funding agency. *Trials* .2019;20(1) : 10.1186/s13063-019-3900-8 714 10.1186/s13063-019-3900-8 31829233 PMC6907219

[2] : Infectious diseases epidemiology, quantitative methodology, and clinical research in the midst of the COVID-19 pandemic: Perspective from a European country. *Contemp Clin Trials* .2020;99: 10.1016/j.cct.2020.106189 106189 10.1016/j.cct.2020.106189 33132155 PMC7581408

[3] : SARS-CoV-2 Cell Entry Depends on ACE2 and TMPRSS2 and Is Blocked by a Clinically Proven Protease Inhibitor. *Cell* .2020;181(2) : 10.1016/j.cell.2020.02.052 271-280.e8 10.1016/j.cell.2020.02.052 32142651 PMC7102627

[4] : TMPRSS2 inhibitors for the treatment of COVID-19 in adults: a systematic review and meta-analysis of nafamostat and camostat mesylate randomised clinical trials. *Clin Microbiol Infect* .2024; 10.1016/j.cmi.2024.01.029 10.1016/j.cmi.2024.01.029 38331253

[5] : The DAWN antivirals trial: process evaluation of a COVID-19 trial in general practice. *BJGP Open* .2023; 10.3399/BJGPO.2023.0109 10.3399/BJGPO.2023.0109 37984980 PMC11300998

# Reviewer report: Reviewer response for version 1

**DOI:** 10.21956/wellcomeopenres.21388.r74109

**Published:** 2024-02-28

**Authors:** Sandro G Viveiros-Rosa

**Affiliations:** 1 National Institute of Industrial Property, Rio de Janeiro, Brazil

Dear Editor and Authors,

     The article is very interesting, well-written, and involves extremely relevant topics in the scientific context regarding the quality of clinical trials, systematic reviews, and the treatments adopted by governments worldwide as a result of these studies.

     When focusing on the need to increase quality in various aspects of clinical trials and systematic reviews carried out in the COVID-19 scenario, the authors also point out several medium and long-term solutions that could be goals for researchers in this scientific segment. 

     Nevertheless, I have some questions that should be reviewed by the authors:

 1. Page 4 (Key Findings chart) - Here the drugs no longer recommended in clinical trials (such as chloroquine and hydroxychloroquine) should be highlighted. In addition, it should be mentioned the approved ones, with more clinical tests, highlighting that remdesivir is expensive but was well tested and approved in many countries, following Table 3. As it is stated in this chart, seems that remdesivir is a doubtable drug with a higher cost that is still given to patients without any sanitary agency around the world approval.

 2. Page 7 and Table 3 - Didn't Ivermectin appear in any of these clinical tests? I think there are a lot of clinical trials with this and for this reason, it should be contained here in Table 3.

 3. Figure 2 - The name of the drug is REMDESIVIR. Need to correct it.

 4. Figure 3 - What does the “ **n”** on the graph mean? Captions are needed here. Furthermore, it would be interesting for this graph to contain more countries at other levels of economic development such as India, Brazil, and China.

 5. Pages 10, 11, and Figure 6 - The authors insist on comparing the cost-effectiveness of two drugs that are not comparable. Dexamethasone is an old corticosteroid while remdesivir is an antiviral formulation, still patented and, for this reason, more expensive. However, remdesivir is approved in several countries and the cost issue could be overcome through agreements between governments and companies or through compulsory licensing by governments. The case of India is quite interesting because the negotiations not only reduced the price but also facilitated access to production and use by its population, as well as the growth of local companies. I think this should be mentioned in the discussion of this chapter. These sources can be used as references: Blair HA, 2020 (Ref 1);  Viveiros Rosa SG et. al., 2022 (Ref 2); Gottlieb RL et. al., 2022 (Ref 3)

 6. Pages 10-12 - Why doesn't the article mention Paxlovid (nirmatrelvir plus ritonavir) in terms of cost-effectiveness and effectiveness if this is the most used and sold antiviral for COVID-19 in the world? It would be very important to compare the cost-effectiveness results between the three best-selling antivirals, Paxlovid, Molnupiravir, and Remdesivir.

Is the review written in accessible language?

Yes

Are all factual statements correct and adequately supported by citations?

Yes

Are the conclusions drawn appropriate in the context of the current research literature?

Yes

Is the topic of the review discussed comprehensively in the context of the current literature?

Yes

Reviewer Expertise:

COVID-19, Zika Virus and Mpox diseases; Techonological Foresight Studies, Patent Reviews, Clinical trials, and Systematic reviews.

I confirm that I have read this submission and believe that I have an appropriate level of expertise to confirm that it is of an acceptable scientific standard.

## References

[1] : Remdesivir: A Review in COVID-19. *Drugs* .2023;83(13) : 10.1007/s40265-023-01926-0 1215-1237 10.1007/s40265-023-01926-0 37589788 PMC10474216

[2] : Remdesivir: an overview of patenting trends, clinical evidence on COVID-19 treatment, pharmacology and chemistry. *Pharm Pat Anal* .2022;11(2) : 10.4155/ppa-2021-0023 57-73 10.4155/ppa-2021-0023 35638315

[3] : Early Remdesivir to Prevent Progression to Severe Covid-19 in Outpatients. *N Engl J Med* .2022;386(4) : 10.1056/NEJMoa2116846 305-315 10.1056/NEJMoa2116846 34937145 PMC8757570
